# Augmented reality for chemistry education to promote the use of chemical terminology in teacher training

**DOI:** 10.3389/fpsyg.2024.1392529

**Published:** 2024-07-22

**Authors:** Melanie Ripsam, Claudia Nerdel

**Affiliations:** Associate Professorship of Life Sciences Education, TUM School of Social Sciences and Technology, Technical University of Munich, Munich, Germany

**Keywords:** augmented reality, AR learning environment, chemical terminology, (multiple) external representations, representation change, substance-particle concept understanding, teacher education and training, split-attention

## Abstract

Chemistry as a whole is divided into three levels. The *macroscopic level* describes real, observable phenomena of the material world. The *submicroscopic level* focuses on particles. The *representative level* includes pictorial and symbolic representations to visualize substance in its nature. Students often have problems separating these levels and conceptually transfer each of the three levels to the other. Therefore, teachers need to use chemical terminology correctly when teaching the substance-particle concept. Augmented Reality (AR) connects real and virtual world. The observer physically moves in a real environment that integrates virtual elements. The AR technology has great potential for learning in the subject chemistry, especially when it comes to making the “invisible” visible and illustrating scientific phenomena at particle level. The simultaneous presentation should avoid split-attention and offers new possibilities to interactively deal with (M)ER. The question arises whether AR has a positive effect on the use of technical language and the associated understanding of the concept of dealing with (M)ER at the substance and particle levels. With an AR app on the tablet and the AR glasses, the chemical processes of a real experiment are represented by AR visualizations. Therefore, the AR app was piloted. This study captured the chemistry handling with (M)ER of chemistry teachers (*N* = 30) using a pre-post survey. The participating preservice teachers are described below. Each test includes five tasks elaborated by thinking aloud. The thinking-aloud protocols to acquire the use of the chemical terminology are evaluated in MAXQDA.

## 1 Introduction

According to Johnstone (2000), chemistry is divided into three levels: (1) The macroscopic level describes real, observable phenomena of the material world. (2) The submicroscopic level focuses on particles such as atoms, ions, molecules, and chemical processes. (3) The representative level includes pictorial and symbolic representations (such as texts, symbols, or images) to visualize substance in its nature macroscopically or submicroscopically. If learners can conceptually transfer each of the three levels to the other, this should positively affect the learning process (Devetak et al., 2004; Farida et al., 2010). Johnstone’s (1993, 2000) three-level model resulted from numerous reflections on the difficulties of teaching and learning chemistry. However, the simultaneous use of the three levels is almost impossible for young learners (Johnstone, 1993). International studies show that students use the particle concept inconsistently and that chemical terminology, with its multiple external representations (M)ER, is challenging (Harrison and Treagust, 2000).

Chemistry teachers, therefore, have a role model function when they teach chemical terminology and the associated modeling processes in addition to the particle concept (Farida et al., 2010; Santos and Arroio, 2016; Rodić et al., 2018). They face the major challenge of helping students to think adequately at the levels, according to Johnstone (1993, 2000) (cf. Santos and Arroio, 2016).

"Unfortunately, most chemistry teaching is focused on the submicro-symbolic pair of the triplet and rarely helps students to build bridges to comfortably move between the three levels." (Talanquer, 2011, p. 181).

However, it also seems complicated for chemistry teachers to learn and teach three-level thinking (Justi and Gilbert, 2002; Van Driel and Verloop, 2002). (Prospective) chemistry teachers have difficulties with correctly using technical language, the substance-particle level change, and the communication of both aspects in the classroom (Justi and Gilbert, 2002; Van Driel and Verloop, 2002; Crawford and Cullin, 2004). According to Johnstone (1993, 2000), thinking in terms of the three levels requires the teacher to use sub-microscopic models to explain the material level (cf. Treagust et al., 2003). However, despite numerous efforts by the teacher to implement these in the classroom, learners do not continually develop an understanding of them. The results of the studies by Treagust et al. (2003) show that students do not always understand the role of representation in illustrating the substance and particle level used by the teacher. In school practice, teachers seem to constantly move between the different forms of chemical representation and, depending on the situation, choose a different, most suitable form of representation to illustrate the substance or particle level. However, this choice is not explained or discussed in detail with the students, leading to difficulties in understanding (cf. Santos and Arroio, 2016). The studies by Devetak et al. (2004) and Al-Balushi (2012) state that teachers rarely orient their teaching concepts toward thinking on all three levels, according to Johnstone (2000), rather they do not adequately link them with each other. In addition, studies with trainee teachers also demonstrate that they lack language skills when explaining substance-particle level changes and do not differentiate sufficiently between the levels (Rodić et al., 2018). They often consider the precise use of technical terms in substance-particle level processes redundant, as they are observed as complications for teaching and learning (Rodić et al., 2018). In view of this, the current findings by Kapici (2023) show that prospective chemistry teachers elaborate most successfully at the symbolic level and make insufficient use of the other levels to explain chemical phenomena or hardly differentiate between them. The results of Farida et al. (2010) provide initial indications that the problems are due to faulty substance-particle level interactions. Prospective chemistry teachers have difficulties explaining processes at the particle level using symbolic representations and understanding the importance of models and drawings at the submicroscopic level (Farida et al., 2010). The pre-post study of prospective primary school teachers (Derman and Ebenezer, 2020) reveals difficulties in using the particle model despite the positive effects of forms of representation to illustrate scientific phenomena on understanding the substance-particle concept. It has been shown that a lack of chemical terminology, in particular, has a negative impact on the understanding of the substance-particle concept (cf. Al-Balushi, 2012). Due to the characteristics of the respective (particle) model and the associated degree of abstraction of the various representations, thinking in the three levels, according to Johnstone (1993, 2000), appears to be both difficult to learn and teach for (prospective) chemistry teachers (Justi and Gilbert, 2002; Van Driel and Verloop, 2002; Eilks, 2012; Santos and Arroio, 2016).

It makes sense to integrate digital media as a supporting measure in subject teacher training (Sailer et al., 2017). Numerous studies are based on the added value of digital media in terms of subject and media didactics, such as animations, and repeatedly confirm that they have great potential for multimedia learning (Mayer and Moreno, 1998, 2002a,b; Sweller, 2011). A benefit should arise from technological advances when visually imperceptible processes are made visible with digital software systems (Farida et al., 2010). Particle modeling techniques (e.g., tablet with video) contribute to understanding the substance-particle concept (Schnitker, 2016). However, in such settings, the viewer is forced to look back and forth between the medium and the real experimental setup. The split-attention effect can disrupt cognitive processing during text-image integration (Schnotz and Bannert, 2003; Ayres and Sweller, 2021). As the working memory capacity is overloaded, learning becomes more difficult (cf. Sweller, 2011). Consequently, the potential of digital media for chemistry lessons cannot be fully exploited. Augmented Reality (AR) links real and virtual worlds (Ibanez and Delgado-Kloos, 2018) so that the observer physically moves in a real environment that integrates virtual elements. In this way, AR enables interaction with real and virtual objects (Azuma, 1997). Using suitable apps on special AR devices, AR objects can be projected into a real environment in the background (see Milgram and Kishino, 1994; Buchner and Freisleben-Teutscher, 2020). After surveying the real world, the camera function on a mobile display device is used to enhance a real image with virtual elements (Milgram and Kishino, 1994). It is possible to view the combination of the physical and digital world in two ways (Milgram and Kishino, 1994), either monitor-based on a single screen (e.g., PC, tablet, smartphone) or via displays integrated directly into the field of vision in the form of AR glasses (e.g., head-mounted display, abbreviated to HMD). However, due to their cost and complexity, the latter is rarely used in everyday life and at school (Wyss et al., 2021). Whereas the user controls the digital objects on the tablet by moving their finger on the screen, the virtual elements, visible through the AR glasses, are moved in space such as real objects. Behavior in the augmented world is similar to that in real life, as the user actually gains the impression of being present in a computer-generated world and adapts their actions accordingly (cf. Slater and Wilbur, 1997). The retrieval of AR using appropriate glasses then creates the feeling of immersion and leads to an immersion in a virtual world, which the individual perceives as an illusion of reality (cf. Slater and Wilbur, 1997; Buchner and Freisleben-Teutscher, 2020). This illusionary experience also entails a different view of the object representations (cf. Ainsworth, 1999; Schnotz and Bannert, 2003). While the AR representation overlays a digitally replicated image of the real world using the camera function on the tablet display, the virtual objects are immersively integrated into the real environment when viewed through the HMD-AR glasses (cf. Buchner and Freisleben-Teutscher, 2020). According to Dunleavy and Dede (2014), AR should be beneficial for constructivist learning environments, as learners are actively involved in the learning process and can control it in a self-regulated manner (Buchner and Freisleben-Teutscher, 2020). The main advantage of AR technology lies in the integration of various static and dynamic (M)ERs into reality (cf. Ainsworth, 1999; Kozma and Russell, 2005; Chavan, 2016), with which the learner can interact as desired (Azuma, 1997). Regarding representational competence, this opens up completely new opportunities for learning in chemistry. In addition to visualizing particles such as electrons, virtual overlays in a real experiment environment can consist of chemical symbols (e.g., reaction equations) or texts (e.g., technical terms) and should be used in a supportive manner depending on the previous knowledge of the viewer (Schnitker, 2016; Akçayır and Akçayır, 2017; Nerdel, 2017). Consequently, AR as an interactive and communicative tool can positively affect the attitudes and motivation of learners and, above all, learning performance (Bacca et al., 2014; Radu and Schneider, 2019). When submicroscopic particles are virtually superimposed on the experiment (while a real experiment is running), the information can be spatially and temporally connected and semantically linked (Chavan, 2016). The technology offers new educational opportunities for multimedia learning (Hellriegel and Čubela, 2018; Buchner and Freisleben-Teutscher, 2020; Keller and Habig, 2022) and can provide promising support for the learning process (Dunleavy and Dede, 2014). From a cognitive psychology perspective, it is conducive to learning to use such interactive visualizations to acquire knowledge (Schnotz and Bannert, 2003; Farida et al., 2010; Mayer, 2014). According to the coherence and contiguity principle of Mayer (2014), Ayres and Sweller (2021) and Fiorella and Mayer (2021) this simultaneous presentation should avoid split-attention and offers new opportunities for successful learning in the levels. However, the number of learning-efficient AR apps for chemistry still appears to be low. The desire for AR applications that enable work with real laboratory equipment, effectively support educational processes, and facilitate learning in chemistry is therefore very high (see Buchner and Freisleben-Teutscher, 2020; Schwanke and Trefzger, 2020; Wyss et al., 2022). Although there are also experimental designs that investigate AR applications as educational technologies in the context of teacher training (cf. Buchner and Zumbach, 2020; Wyss et al., 2021, 2022) and often show initial tendencies of positive effects on motivation and learning success (cf. Buchner and Zumbach, 2020), studies on AR technologies in teaching-learning situations should nevertheless be regarded as a research desideratum (cf. Wyss et al., 2021).

### 1.1 Aim and scientific questions

Due to its technical functions, AR offers the best prerequisites for being used as a support measure to promote the handling with (M)ER at the substance and particle levels in real experiments. In addition to the temporal and spatial integration of AR objects into a real experimental setup, AR enables interactivity with the augmented forms of representation, such as the sub-microscopic particles, and does not ignore the dynamics. Thus, this study focuses on the learning effectiveness of an AR learning environment (on a tablet or AR glasses) to promote the use of chemical terminology, i.e., dealing with (M)ER, among chemistry teachers. Therefore, a learning environment was designed to expand the professional knowledge of teachers. The target is to be able to use innovative digital technologies in the subject lessons with students in perspective and didactically reflected way.

Accordingly, it is assumed that AR settings improve the handling of the forms of representation or chemical terminology and thinking in the three levels, following Johnstone (1993, 2000). From a cognitive psychology perspective, AR learning environments are superior to other digital learning environments because they adhere to the design criteria of coherence and contiguity, avoid split-attention, and can therefore initiate mental modeling processes to improve the understanding of substance-particle concepts. As a result, it is expected that after working through the non-AR learning environment, misconceptions will only be reduced to a limited extent or even remain constant. About dealing with the forms of representation and chemical terminology, only a small positive change is assumed. This leads to the following research question with the hypothesis:

**RQ1**: Can the AR learning environment promote reflective use of technical language at the substance and particle level from a teaching perspective among chemistry teachers (AR vs. non-AR)?

*H1*: It is hypothesized that the use of an AR learning environment promotes the integration of the representation level when observing a real experiment and improves, in this context, the substance-particle concept understanding. Using the simulation should disrupt cognitive processing and improve chemical terminology to a much lesser extent. By avoiding split-attention, AR is expected to support the construction of mental models and thus largely shape elaboration behavior.

The positive influence of AR should become particularly apparent after processing the HMD-AR learning environment. It is reasonable to assume that interactivity with the AR representations when wearing AR glasses positively affects the use of chemical terminology and visibly improves representational competence (Kozma and Russell, 1997, 2005). Finally, the immersion of the real and virtual world, i.e., the strongly pronounced reality, is more than just motivating (Wyss et al., 2021); the linking of real objects with immersive AR representations should also make it easier to operate on a representational level and thus counteract cognitive overload (cf. Sweller, 2011; Schnotz, 2014). It is therefore expected that test subjects who use the HMD-AR technology will react more sensitively to the interactivity, and that, their thinking on the three levels, according to Johnstone (1993, 2000), will be immensely supported as a result. The following research question is therefore derived from the corresponding hypothesis:

**RQ2**: Can the interactive use of (immersive) augmented representational forms in the learning environment, with regard to the use of tablet or AR glasses (AR vs. HMD-AR), describe different elaboration profiles?

*H2*: It is hypothesized that using the AR learning environment on a tablet, especially on AR glasses, has a positive effect on the use of chemical terminology. The simultaneous linking of HMD-AR representations with the content of the real experimental environment is expected to initiate cognitive processing. In addition to this, (M)ER can be controlled in a self-regulated manner. Different elaboration profiles are expected when interacting with augmented (M)ER on the tablet *or* AR glasses.

## 2 Methods

### 2.1 Participants

The subjects are teachers from German secondary schools (65% women and 35% men; age *M* = 28, SD  =  5.2) who teach chemistry (*N* = 30). Over half of the teachers have been in service for at least 6 years. According to Hubermann (1991), they can therefore be regarded as (very) experienced teachers. All other test subjects have been working as teachers for 4–6 years and are, therefore, in a stabilization phase, which indicates a moderate to slightly increased level of professional experience. All test subjects stated that they use digital media privately, for example, for communication or entertainment purposes (“social media” or “YouTube videos”) and also regularly incorporate these into their lessons. Only one respondent said he/she would use AR privately (e.g., “PokemonGo” from the gamefication sector) experimental group 1 consists of 10 subjects working with an AR learning environment on the tablet, and experimental group 2 works with the same AR learning environment on AR glasses. The control group comprises 10 other subjects working with a content equivalent simulation-based learning environment on the tablet. This results in two cell populations of 10 and 10 test subjects, which is sufficient according to a qualitative sampling plan (Döring and Bortz, 2016).

### 2.2 Experimental designs

The experimental study to analyze the influence of AR on the use of chemical terminology is based on a single-factor pre-post design (see Table 1). The independent variable (IV1) of study design 1 consists of the media and instructional design of the learning environment and has two characteristics. The user control in the AR learning environment (simulation-based learning environment) is varied by using virtual forms of presentation (animated forms of presentation). Both learning environments can be called up on the tablet medium and are identical regarding content. While the simulation creates split-attention, as the data are not linked to the real experimental apparatus regarding time and space, AR ensures contiguity in its integrated format. The dependent variable (DV) is handling (M)ER, which is operationalized by the adequate change between the representations on a macroscopic and submicroscopic level. Based on the findings of cognitive psychology on the split-attention effect according to Ayres and Sweller (2021), the contiguity principle according to Fiorella and Mayer (2021) stated that differences are expected between the experimental group (*N* = 10), which works with AR, and the comparison group (*N* = 10), which elaborates the simulation (cf. H1 in Chapter 1.1).

**Table 1:** Study designs 1 and 2; IV1 Media and instructional design of the learning environment and IV2 Interactivity in the AR learning environment and the expected effects on DV Dealing with (M)ER.

	IV1: Media and instructional design of the learning environment	
	Control group *Simulation on tablet* (*N* = 10)	Experimental group *AR learning environment on tablet* (*N* = 10)
DV: Dealing with (M)ER	Slight improvement in the use of forms of representation and technical aspects at the substance and particle level of language.	Significant improvement in the use of forms of representation and technical language at the substance and particle level

	IV2: Interactivity in the AR learning environment	
	Control group *AR learning environment on tablet* (*N* = 10)	Experimental group *AR learning environment on HMD-AR* (*N* = 10)
DV: Dealing with (M)ER	Significant improvement in the use of forms of representation and technical language at the substance and particle level	Visibly improved handling of forms of representation and technical language at the substance and particle level.

In addition, it should be investigated whether the interactivity of the AR learning environments, due to the immersive characteristic of the AR setting, has a positive influence on handling (M)ER (cf. H2 in Chapter 1.1). The study, therefore, used a further single-factorial design with pairwise group comparison (see Table 1). Accordingly, interactivity in the AR learning environment was considered the second independent variable (IV2). It is based on two characteristics: On the one hand, the AR representations can be viewed and controlled interactively on the tablet screen. On the other hand, using HMD-AR technology on the AR glasses enables interactive control of the virtual representations in the natural environment with an immersive experience. The learning environments with the same content are now subjected to a change of medium. By the experimental design presented above, the experimental group once described, which accessed AR on the tablet, now mutated into a control group (*N* = 10). This was compared with a new experimental group of 10 additional participants working with the HMD-AR learning environment on the AR glasses. Analogously, the change position of (DV) was operationalized as the dependent variable. Significant effects are expected in the experimental group working with AR on the tablet about the influence of IV2 on DV. Finally, the experimental group using the AR glasses should show an even more significant effect of IV2 on DV, as they can operate better on a representational level with the immersive augmented (M)ER. This should immensely enhance the use of chemical terminology.

### 2.3 Design of the AR learning environment

The AR learning environment on the subject of redox reactions consists of a real experimental setup for the electrolysis of zinc iodide. The virtual learning environment appears in the foreground as soon as the subjects point a tablet/look through AR glasses with the application at the electrolysis cell (Chavan, 2016). AR glasses are rarely or hardly ever used in school lessons (Tschiersch et al., 2021), so the (non-)AR learning environment was also transferred to an HMD-AR variant. The function menu can interactively direct which (M)ER is virtually projected onto the real experiment (Schmalstieg and Höllerer, 2016). AR learning environments can be designed in a variety of ways with regard to pedagogical and didactic approaches and offer various individualization options to promote the acquisition of knowledge and skills in different ways (Anderson and Anderson, 2019; Garzón and Acevedo, 2019). The AR learning environment in the research project was designed based on the model for the development of a digital learning environment for mathematics lessons, according to Reinhold (2019), in order to validly record the use of chemical terminology at the substance and particle level using AR. Attention was paid to the four core elements of “subject content,” “support focus,” “design,” and “usability”:

#### 2.3.1 Subject content

The chemical subject knowledge plays an important role in the research project and should not represent an additional challenge for the processing of the learning environment. The donor–acceptor concept for electron transitions can be found in the concept of chemical reactions section of the chemistry subject profile for the ninth grade of grammar school in Bavaria (cf. Staatsinstitut für Schulqualität und Bildungsforschung, 2023a). As an essentially harmless chemical experiment, it is regularly practiced in the chemistry classrooms of secondary and grammar schools. The learning environment should expand or explain the real chemical phenomenon using AR aids and promote scientific work with the real object (cf. Klos et al., 2008). The real experimental setup is not replaced by the technology (cf. Bacca et al., 2014) but merely supplemented meaningfully (cf. Goldkuhle, 1993). Given this, the choice fell on the electrolysis of zinc iodide experiment in the chemistry lessons.

#### 2.3.2 Support focus

Most AR teaching and learning tools for STEM lessons are based on exploratory or simulation-based applications (Ibanez and Delgado-Kloos, 2018). The AR learning environment of the research project described is also set up on the basis of a simulation. The unique feature of the AR setting described is based on the integration of actual laboratory equipment, which, however, has rarely been used in AR-supported learning scenarios (cf. Buchner and Freisleben-Teutscher, 2020; Schwanke and Trefzger, 2020; Wyss et al., 2022). If the framework conditions and subject content of the AR setting are combined, the focus is mainly on the substance-particle level change through the use of (M)ER. Accordingly, the actual experimental setup represents the material world, which is enriched with explanations on the submicroscopic level using virtual objects. Accordingly, the real phenomenon of electrolysis of zinc iodide is to be identified as a substance level, and the modeled AR objects in the learning environment are to be interpreted on a submicroscopic and representative level (cf. Johnstone, 2000). It is necessary for the levels to be viewed in a differentiated manner from one another and to be able to be transferred into one another (cf. Taber, 2013; Reid, 2021). Supplementary AR displays provide additional information on both levels and guide the user through the learning environment. The AR learning environment is designed to facilitate thinking at the three levels, according to Johnstone (2000). Based on the didactic study by Keller and Habig (2022), Kuhn et al. (2017), Schwanke and Trefzger (2020), or Thyssen et al. (2020), aspects such as the spatial imagination of 3D modeling and scaffolding in scientific work were taken into account when designing the AR learning environment.

#### 2.3.3 Design

The setting is based on the valuable findings of cognitive psychology (cf. Schnotz and Bannert, 2003; Fiorella and Mayer, 2021). In line with cognitive load theory, the setting should, therefore, be designed as simple as possible and only as detailed as necessary in order to counteract unnecessary cognitive load through the design of the learning environment (cf. Extraneous Cognitive Load according to Kalyuga and Sweller, 2014; Mayer, 2014). As a result, the learning environment was structured using learning paths. Finally, the technical clarification (support focus) and the learner perspectives (cf. difficulties with chemical terminology; Vosniadou, 1994; Kapici, 2023) were equally integrated into the conceptual development process of the learning environment. With the help of AR, a new possibility of didactic structuring (cf. Reinfried et al., 2009) for dealing with (M)ER at the substance and particle level was to be achieved. Based on the model of multimedia learning according to Mayer (2002), the type, number, arrangement, and linking of the forms of representation in the learning environment were thoroughly investigated. The AR learning environment has the most significant special feature about the coupling of the AR objects with the process in the real test apparatus. Following the coherence and contiguity principle of Fiorella and Mayer (2021), the virtual, submicroscopic models were linked spatially and temporally with the real, observable chemical experiment, taking into account the reaction dynamics, so that the information is semantically related (cf. Chavan, 2016; Schmalstieg and Höllerer, 2016). To adapt the contents of the AR learning environment to the needs and previous knowledge of the learners, a manageable set of ions was chosen. Cognitive load (cf. Sweller et al., 1990) was thus to be avoided. In particle modeling, attention was paid to ion size ratios and atomic and molecular radii, but their diameters or radii were not specified numerically. Since electrolysis and diffusion are already two significant, extensive chemical topics, dissociation was not directly integrated. A help button can be clicked to get information about the hydrate sleeves.

#### 2.3.4 Usability

In addition to the design criteria listed above, the technical and conceptual implementation was based on the principles of EN ISO 9241-110 (Prümper, 2008; Figl, 2010) and the design criteria of Kopp et al. (2003). The AR setting includes four learning paths elaborated before and after the DC source is turned on: *Experimental Setup*, *Diffusion*, and *Electrolysis at the Particle Level* and *Chemical Reactions*. Within a learning path, concrete changes in the presentation were integrated in terms of content: The user can distinguish between the presentation forms *text, symbol*, and *image*. For example, the principle of controllability is emphasized by the adaptive selection options of the learning paths with associated forms of representation (text, symbol, and image) (cf. Bannert, 2009). The learning environment should not only be based on AR-supported aids to promote self-regulated learning (cf. Huwer et al., 2019; Fleischer et al., 2022) but also enable new ways of working with virtually (M)ER in real experiments. Accordingly, care was taken to ensure that the test subjects could decide for themselves as far as possible which learning paths and the information contained (e.g., first reduction and second oxidation or vice versa) should be projected onto the real object. Furthermore, the authentic design of the particle processes in the typical experiment “Electrolysis of zinc iodide” can be subordinated to the design criterion of problem-oriented didactics (cf. Bürg, 2005).

**Figure 1:**
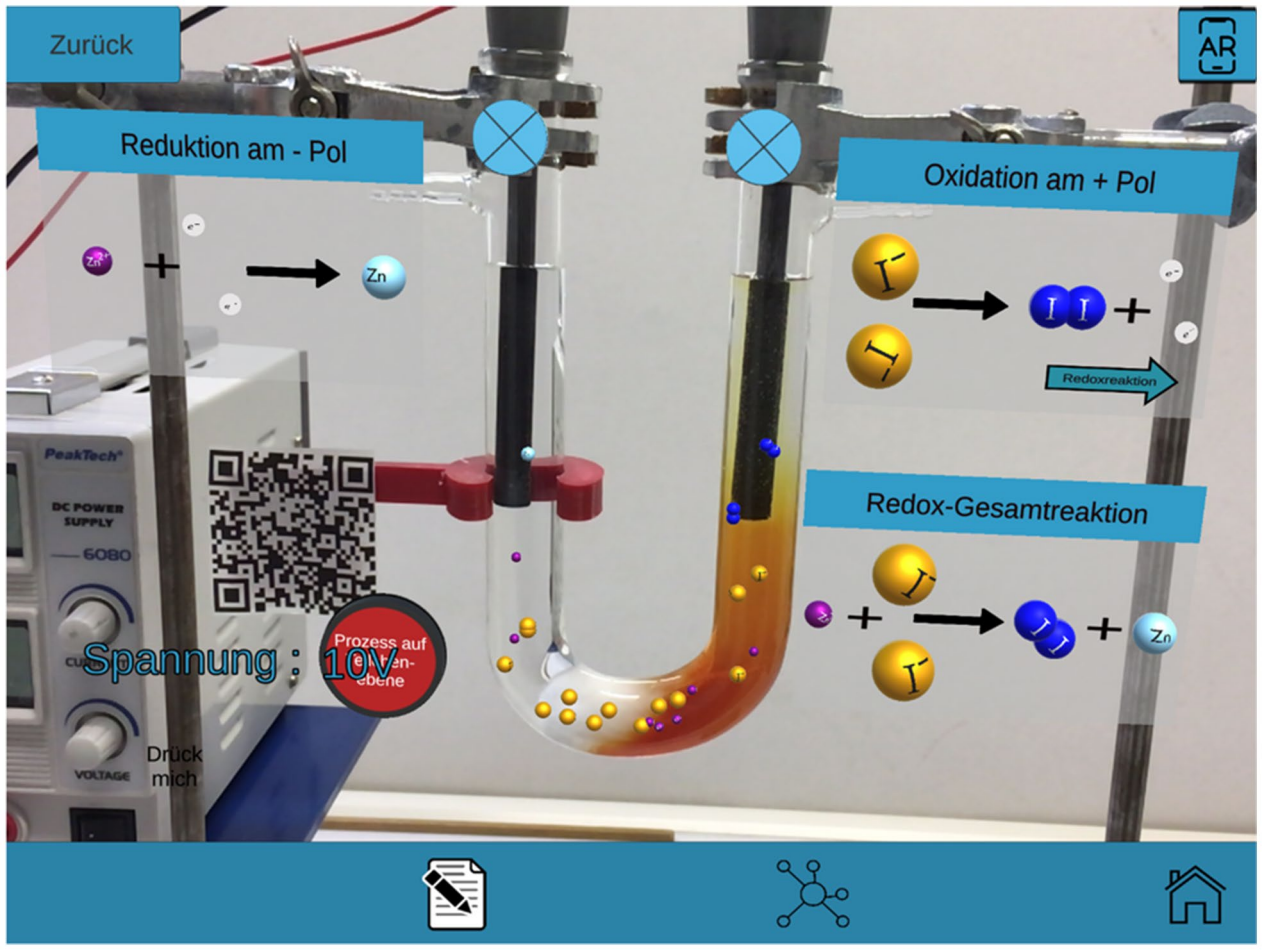
View through a tablet on the real experiment with virtual overlays of the learning path. *Chemical reaction*: Pictorial representation after clicking all buttons with the particle level processes (after switching on the DC voltage source).

Figure 1 exemplifies that the user can view the chemical reactions pictorially and simultaneously project the particle-level processes into the real experiment. The particle-level processes are always oriented to the real experiment sequence at the substance level (Azuma, 1997).

The design criteria above were considered when developing the three learning environments. However, regarding interactivity when using AR glasses, the programming of the HMD-AR technology played a unique role and was associated with increased programming effort (see Buchner and Zumbach, 2020; Wyss et al., 2021).

### 2.4 Survey instruments and method

#### 2.4.1 Questionnaire

Two topic-specific tests on the donor–acceptor concept were created to record the handling of (M)ER (see DV in Chapter 2.2; Table 1). The two test instruments were used as pre- and post-tests before and after processing the respective learning environment (cf. Jonkisz et al., 2012). To analyze the effect of AR on dealing with (M)ER and the related construction of mental models about redox reactions, the method of thinking aloud is used. For this purpose, subjects’ utterances are recorded while processing the test tasks.

Table 2 provides an overview of the pre-test and post-test items with their respective structures.

**Table 2:** Overview of task classification in pre- and post-test about type, subject content, representation changes, and anchor tasks.

	Task	Type	Content	Change of representation		Anchor task
				From	In	
*Pre-test*	1	Open	Redox reaction synthesis NaCl	text	text + symbol + picture	
	2	Open	Acid–base reaction neutralization	(text) + symbol	text + symbol	
	3	Open	Redox reaction galvanizing	text	text + symbol + picture	X
	4	Open	Redox reaction extraction of lead	text + (symbol)	text + symbol	X
	5	MC	Redox reaction mercury/nitrate	text + symbol	text + symbol	
*Post-test*	1	Open	Acid–base reaction cyanidin	Text + symbol	Text + symbol	
	2	*See above anchor task 4 in the pre-test*				
	3	Open	Redox reaction blast furnace process	text + (symbol)	text + symbol + picture	
	4	*See above anchor task 3 in the pre-test*				
	5	MC	Redox reaction copper/iron	text + (symbol)	text + symbol	

The parenthesis “()” demonstrates the relatively lower use of the representation form in the task.

To be able to record the effectiveness of AR in dealing with (M)ER, attention was paid to quality parameters during test construction. All tasks from the pre-test and post-test were designed to provoke various directions of cognitive processing in the chemistry teachers by means of elaboration by thinking aloud (Sandmann et al., 2002). On the one hand, knowledge retrieval from memory and, on the other hand, knowledge building through knowledge generation using (logical) inferences should be initiated (Kintsch, 1993; see category system in Chapter 4.1.

Each test comprises five self-created test tasks on the chemical donor–acceptor concept (cf. Kultusministerkonferenz, 2005; Staatsinstitut für Schulqualität und Bildungsforschung, 2023a), which are intended to describe and explain chemical phenomena, whereby translation skills between the (M)ER are specifically enforced. They always focus on constructing, interpreting, and translating (M)ER. Table 2 shows that both tests have an identical structure in terms of the tasks’ number, type, and subject content. Furthermore, two anchor items were integrated by including two test items from the pre-test and the post-test without any changes in content or form (cf. Walpuski and Ropohl, 2014). The questions of the open-ended and MC tasks consist of the task base and the answer format (cf. Rost, 2004). The task base always contains a chemical question or problem for which a solution must be developed. The tasks were designed in such a way that they require didactic justifications based on the subject content. For implementation objectivity, clear work instructions were integrated into all test tasks, which were formulated in precise language (cf. Lienert and Raatz, 1998; Jonkisz et al., 2012). In general, identical translation performance is expected in both tests, as changes of representation from text (and symbol) to text and symbol (and picture) can always be achieved (see Table 2). However, these are more complex to implement in the post-test. To achieve a high degree of test quality, 13 tasks, including those from the pre- and post-tests, were analyzed by eight subject didactics experts with experience in item development (cf. Osterlind, 1998; Terzer et al., 2013). This was followed by a trial test run with all tasks and an expert rating to assess the possible elaborations, based on which the tasks were evaluated, selected, and further developed about their quality (cf. Tepner and Dollny, 2014).

#### 2.4.2 Data collection

**Figure 2:**
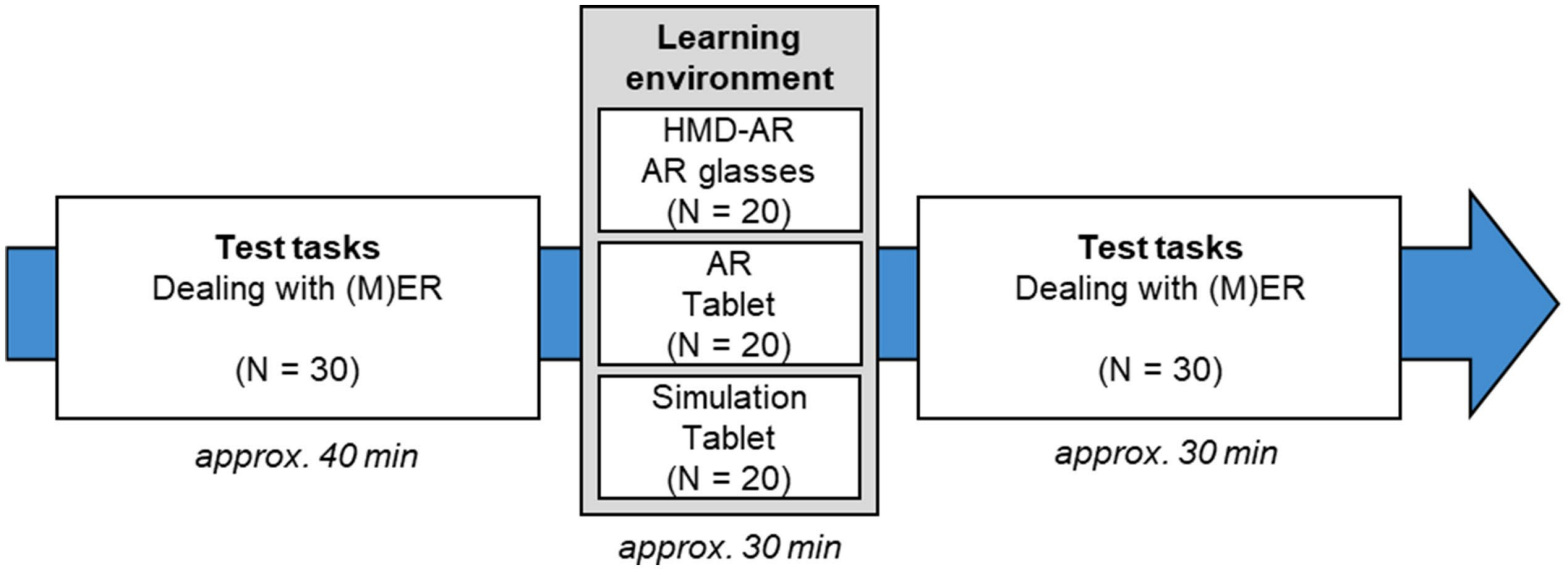
Implementation and procedure of the data collection: Recording the use of (M)ER and processing one of the three learning environments.

To investigate technical language and the associated understanding of the concept of dealing with (M)ER at substance and particle levels via the elaboration behavior of virtual representations in the AR environment among chemistry teachers (see Chapter 1.1; Research Questions 1 and 2), all subjects in the study participate in a pre-post survey (see Figure 2). Before starting the learning environments, subjects are informed about what they need to pay attention to when completing the tests and thinking aloud. This is followed by the completion of the post-test. Teachers are then briefly instructed on using the digital device (AR or simulation-based technology) on the tablet/AR glasses. Afterward, the experimental group 1 resp. 2 works on the AR learning environment on the tablet resp. AR glasses. The control group works on the simulation-based learning environment on the tablet. The simulation-based learning environment is designed to be similar in content to the AR environment but has, compared with the AR app, on the tablet a detrimental split-attention effect from a cognitive psychology perspective (Azuma, 1997; Mayer, 2014; Schnitker, 2016). During the interaction with the AR-App or simulation, subjects are asked to describe the experiment thinking aloud and explain the process at the particle level with (M)ER (cf. Chapter 2.3). The post-test to assess the understanding of the handling with (M)ER concludes the data collection (see Research Questions in Chapter 1.1).

#### 2.4.3 Analysis methods

To analyze the effect of AR on dealing with (M)ER (see DV, Table 1 in Chapter 2.2) the method of thinking aloud from cognitive psychology was applied (Ericsson and Simon, 1993). For this purpose, the verbal utterances of the test subjects were recorded while they were working on the test tasks. Thinking aloud can depict thought processes, solutions and processing strategies, suggestions, ideas, knowledge content, feelings, perceptions, and sensations of thinking test subjects during an action (Sandmann, 2014). This research method is intended to generate a large amount of data material (Rost, 1998), which provides access to the thought processes during the elaboration of the test tasks (Sandmann, 2014). The thinking-aloud protocols of processing the test tasks will be analyzed with qualitative content analysis, according to Mayring (2010), until the category system is fully validated. Therefore, the statements will be transcribed (Bortz and Döring, 2006). The categorization and coding of the transcripts will be done with MAXQDA. A category system based on the study by Kroß and Lind (2001) will be used for the qualitative analysis. The category system is based on five main categories, which always differentiate between text, symbol, and image. In this context, inferences (e.g., building a situation model) should be recorded mainly (cf. Lind et al., 2005). This will capture whether types (Mayring, 2010) emerge regarding elaboration in the AR (un)supported learning environment. After deductive category formation, the category system will be inductively finalized by analyzing the data material. In total, 20–25% of the data material is double-coded by two independent raters to assess the appropriateness of the categorization (Bortz and Döring, 2006). Quantitative coding of the (primary) categories (Wirtz, 2013) aggregates the data. By determining frequencies of individual trait expressions, trait profiles of the subjects will be obtained (see Chapter 1.1).

## 3 Pilot study for the evaluation of the AR learning environment

Before the AR learning environment could be used in the study, its quality had to be tested. If AR is to be applied in the classroom, the teachers must accept the teaching and learning offer (Bürg, 2005). Acceptance models for information systems, such as the TAM model, from the Anglo-American world deal with the perceived benefits and ease of use, which have an effect on acceptance (cf. Davis, 1989; Goodhue, 1995; Venkatesh and Davis, 2000). Model extensions also include personal characteristics as social and cognitive-instrumental process variables (cf. Venkatesh and Davis, 2000; Kopp et al., 2003) as well as characteristics of the learning environment as influencing factors (cf. model for knowledge media by Simon, 2001; Kopp et al., 2003). Acceptance requires an upbeat assessment of the information/system quality of the innovation (content and characteristics of the learning environment/usability) by the target group (Figl, 2010). Therefore, the pilot study examined how science experts evaluate the features of the AR learning environment (usability) and to what extent they accept the learning environment.

### 3.1 Participants

In March 2021, the AR learning environment (acceptance/usability) review took place (*N* = 18). Natural scientists, (prospective) chemistry teachers, science educators, and software developers were interviewed, all of whom use digital media regularly. Half of the subjects consisted of teachers.

### 3.2 Materials

The task was to pilot the beta version of the AR learning environment. This was the setting described conceptually in Chapter 2.3. At the time of piloting, it was a simplified layout with navigation through the learning paths, that was not intuitive enough. Furthermore, the programming of the perspective changes (e.g., change position of the tablet/zoom into the U-tube) had not been completed. Help buttons were missing and particle modeling was undeveloped.

### 3.3 Procedure

All subjects engaged with the AR learning environment using a tablet. During the interaction with the AR learning environment, the participants had to explain the processes on the particle level with different representations. Subsequently, the questionnaire on the acceptance and usability of the AR learning environment was completed by the subjects.

### 3.4 Questionnaire

During the piloting of the AR learning environment, scales according to Kopp et al. (2003) on acceptance, assessment of didactic and media-didactic design criteria, technical facilitation of learning, learning process, and anticipated learning success/learning transfer are used to investigate the suitability of the AR learning environment against the backdrop of research questions 1 and 2 (see Chapter 1.1). Questionnaire development was also based on previous studies by Bürg (2005); Prümper (2008) and Wolf and Söbke (2020).

### 3.5 Results

A reliability analysis of the AR learning environment provided predominantly good to excellent internal consistency values:

The Acceptance scale (example item: “*I would use the AR learning environment in my own chemistry classes.*”) with seven items has Cronbach’s alpha of .73. The eight usability scales (example item: “*The AR learning environment is likely to spark learners’ curiosity about redox reactions at the material and particle levels.*”) with 4 to 17 items per scale also show Cronbach’s alpha between .668 and .904. Furthermore, all scales on the characteristics of the learning environment have mean values above the mean scale level (see Table 3).

**Table 3:** Descriptive statistics and quality of the 9 scales from questionnaire on a four-point Likert scale from 0 = I do not agree to 3 = I agree completely; number of items (*N*), mean values (*M*), standard deviation (SD) and Cronbach’s alpha (*α*) are given.

Scales	*N*	*M*	SD	*α*
Acceptance	7	2.35	2.99	.737
Instructional support	6	1.88	2.91	.668
Technical usability	9	2.19	4.40	.764
Individualization	4	2.35	1.88	.669
Problem-oriented didactics	17	2.32	6.49	.885
Comprehensibility of media	12	2.48	5.11	.827
Media effect	15	2.38	5.84	.828
Learning process: Anticipated motivation	6	2.31	2.95	.874
Learning process: Expected learning success	17	2.20	8.36	.904

### 3.6 Discussion and outlook

In our pilot study, the conception of the learning environment, despite small flaws, is rated very positively. This positive assessment of usability provides first indications that the setting is accepted by the subjects. Based on the pilot results, the AR learning environment and test instruments were optimized (cf. final versions in Chapter 2.3 and Chapter 2.4.1) to be used in the main study.

## 4 Results

### 4.1 Final category system for dealing with chemical terminology (CAT) with exemplary evidence

A total of 6,105 subject statements were categorized in CAT across all three comparison groups, which could be taken from the 60 pre-and post-test transcripts. Of these, 2,121, 1,930, and 2,054 statements were attributable to the AR, simulation, and HMD-AR groups. Since handling the (M)ER is to be recorded in its entirety, CAT had to delve deeply into understanding the text image. As expected, the coding was based on the theoretical constructs according to Kroß and Lind (2001; see Chapter 2.4.1 and Chapter 2.4.3. Active chemistry teachers should reveal a variety of technical language expressions at substance and particle levels that reveal information retrieval, construct-related integration, and translations from different representations (cf. Kozma and Russell, 2005). Consequently, a distinction is made between the forms of representation of the test tasks (text and symbol). Texts include technical terms or compound or trivial names, symbols of various elements, coefficients, phase symbols, and formula types such as structural, particle, or summation formulas, as well as their reaction equations and MER the combination of both. Overall, 20% of the data material using (M)ER was double-coded. Cohen’s κ of .89 was determined, indicating a high and satisfactory inter-coder reliability. The final CAT can be broken down into three main categories, which should enable a differentiated coding of statements regarding chemical terminology. The three main categories can be divided into nine subcategories, with 36 subcategories (see Table 4 below with selected anchor examples).

**Table 4:** Overview of the main and subcategories of CAT for dealing with (M)ER based on their inductive categories; with final numbers of subcategories.

Main or subcategory	Number of subcategories	Selected Anchor example
1. Adding knowledge elements to the recall of related knowledge from memory 1.1. Mention of knowledge elements that are not included in the task being dealt with 1.2. Search for relationships 1.3. Check/read again	∑ = 7 3	*“Where do I see a cation now? In principle, that would be completely on the left side here.”* (Cat. 1.2.2 Search for relationships with the symbol; post-test; test person 29; simulation group)
	3	
	1	
3. Adding knowledge by generating knowledge using (logical) inferences 3.1. Inferences (text basis/mental conception) 3.1.1. Paraphrasing 3.1.2. Establishing relationships 3.1.3. Describing solutions	∑ = 23 15	*“So at the substance level, a salt is reacted with carbon.”* (Cat. 3.1.1.4 Paraphrasing from symbol to text; post-test; test person 21; simulation group)
3.2. Inferences (situation model)	6	
3.3. Further inferences	2	
4. Reduction of detailed knowledge through deletion	∑ = 6	*“So you would leave out the term reduction here for the time being.”* (Cat. 4.2.1 Reduction text; pre-test; respondent 19; AR group)
4.2. Focus on the main points	3	
4.3. Reducing details	2	
4.4. Text-symbol summary	1	

#### 4.1.1 Main category 1: adding knowledge elements by retrieving related knowledge from memory

Based on Kroß and Lind’s (2001) category of adding knowledge by retrieving related knowledge, a main category could be derived, which is based on the concept of “search-oriented learning,” according to Schmalhofer (1996). It subsumes utterances attributed to the (un-)successful recall of previous knowledge. Accordingly, the respondent can attempt to reconstruct knowledge from memory and recreate it with the help of external sources of information. Category 1 includes searching for relationships between the ER showed in the task at the substance and particle levels, which may fail. When dealing with (M)ER, it is assumed that the knowledge retrieval or search is based either on the texts or symbols of the tasks. In contrast to Kroß and Lind (2001), category 2 on unsuccessful knowledge retrieval was not included separately but in this category of CAT.

#### 4.1.2 Main Category 3: addition of knowledge through knowledge generation using (logical) inferences

The main classification features of this category, based on the taxonomy of inferences in text comprehension according to Kintsch (1993) and following Guthke and Beyer (1992), are the development of a textbase and a situation model (Kroß and Lind, 2001). According to Schmalhofer’s (1996) “understanding-oriented learning,” the application of this type of elaboration differentiates between “superficial” and “deep” understanding (Lind et al., 2004). Following this, the processes of superficial comprehension are characterized by inferences that include the development of a visual image and a textual basis. They are based on the external forms of representation *text* or *symbol* and, therefore, involve paraphrasing, establishing relationships, and describing solutions against the background of dealing with (M)ER (Lind et al., 2004). They initiate “deep” understanding, which is aimed at inferences that integrate the depicted (M)ER at the substance and particle levels into one’s previous knowledge (Van Dijk and Kintsch, 1983). This, in turn, leads to the independent construction of a situation model (Lind et al., 2004). Given this, solution strategies based on conclusions, results, self-generated sub-problems, or diagnoses of own errors should be uncovered during elaboration (Kroß and Lind, 2001; Lind et al., 2005). Furthermore, category 3 focuses on inferences that go beyond the content given in the task and the use of (M)ER (e.g., comments on the methodological procedure or doubts).

#### 4.1.3 Main Category 4: reduction in detailed knowledge through deletion

The category for reducing detailed knowledge is intended to extract the main points. According to Kroß and Lind (2001), information should be removed from the given database by deleting irrelevant details and, as a result, stating that they are unimportant. In the evaluation, this coding should be examined closely, as they affect the development of solutions and can justify strengths and weaknesses in chemical terminology (Table 4).

### 4.2 Group comparisons to investigate the effectiveness of AR on the use of chemical terminology

#### 4.2.1 Impact of the media and instructional design of the learning environment

The evaluation of the coding with CAT resulted in 2,121 statements from the 10 participants in the AR group, which could be assigned to the two measurement times. Of these, 1,106 subject statements were made in the pre-test and 1,015 in the post-test. For the 10 subjects in the simulation group, 1,930 statements were categorized, of which 970 were assigned to the pre-test and 960 to the post-test. The group differences in statement frequencies were minor in the main categories. The absolute frequencies of the pre-test indicate that the two comparison groups had very similar prerequisites for dealing with (M)ER. The assessment of the categorizations from the first to the second measurement point makes it clear that a decrease in statements in category 1 relating to “addiction-oriented understanding” (Schmalhofer, 1996) could be diagnosed in both groups. At the same time, an increase in coding in category 3 relating to “understanding-oriented learning” (Schmalhofer, 1996) was measured in the AR and the simulation group. Although the number of statements in category 4 decreased from the first to the second measurement time point in the AR group and increased in the simulation group, the differences are minimal.

##### 4.2.2.1 Main category 1: adding knowledge elements by retrieving related knowledge from memory

**Mention of knowledge elements**: Subcategory 1.1 *mention of knowledge elements not dealt with in the task just dealt with* shows a reduced number of codings by 7.33% in the AR group from pre-test to post-test. In contrast, the simulation group made more statements, increasing by 7.03%. The statements of both groups are primarily based on the retrieval of information from previous knowledge, which decreased from pre-test to post-test in both groups, especially in the simulation group, and shifted primarily to the naming of knowledge elements from the learning environment.

**Search for relationships**: In addition, fewer searches for relationships were made in both groups from measurement time 1 to 2.

**Figure 3:**
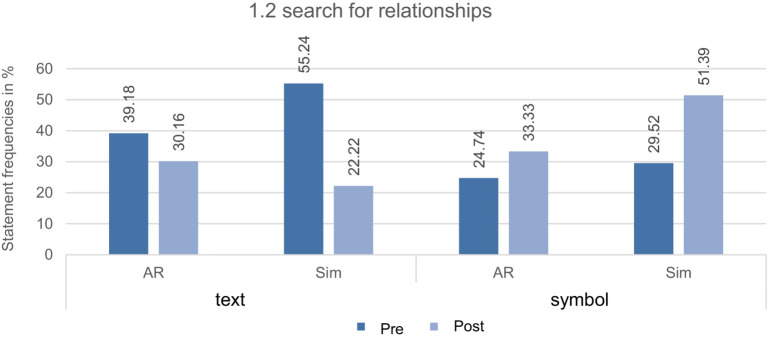
Overview of the relative statement frequencies in percent from CAT of the AR and simulation group (AR and Sim) at measurement times 1 and 2 for the subcategories of 1.2 search for relationships with their population of statements from pre-and post-test (*N*_Pre_ = 202, *N*_post_ = 135).

Figure 3 demonstrates a similar trend in both groups, in which the participants searched for relationships less with texts and more with symbols of the tasks after the treatment. Accordingly, a decrease of 9.02% was measured for the texts and an increase of 8.59% for the symbols in the AR group. In comparison, significantly higher percentage differences of 33.02% were diagnosed regarding the texts and 21.87% regarding the symbols in the simulation group. Overall, the data indicate that the treatment primarily produced behavioral developments about addiction-oriented learning in the simulation group. The qualitative content analysis showed that the general elaboration behavior has changed when comparing measurement times 1 and 2 across both groups. The anchor example below demonstrates that the participants in the pre-test searched for relationships with the texts to establish references at the substance or particle level. In doing so, they focused on terms such as “aquatized” for the separation of the levels (cf. below anchor example^1^; task 5; pre-test):

"I can't quite classify this aquatized state, whether you assign it to the substance or the particle level. But you have to say that aggregate states are properties. Or no, they are not properties, but you assign them to the particle level. Because the particles are - no, the aggregate states are at the material level. […]." (respondent 25, simulation group, subcategory 1.2.1 search for relationships with the text).

##### 4.2.1.2 Main category 3: adding knowledge by generating knowledge through inferences

The group comparison regarding the coding of 3.1 *inferences that build up a text base or visual image* shows minimal differences, whereby a statement increase was measured in both groups after the treatment. However, in the post-test, fewer statements from the simulation group could be assigned to this sub-category, whereas the AR group made more statements regarding “superficial learning” overall.

**Figure 4:**
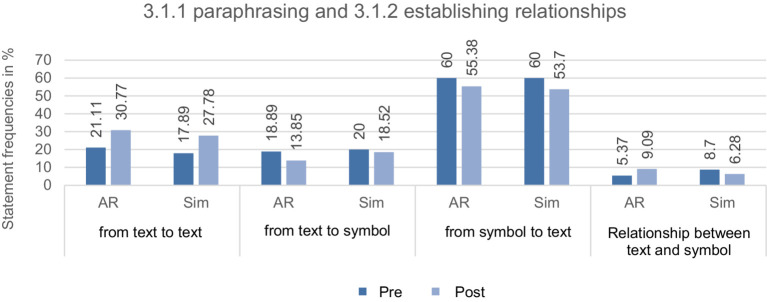
Overview of the relative statement frequencies in percent from CAT of the AR and simulation group (AR and Sim) at measurement time 1 and 2 for the subcategories of 3.1.1 paraphrasing and 3.1.2 establishing relationships with their populations of statements from pre-and post-test (category 3.1.1: *N*_Pre_ = 185, *N*_Post_ = 119 und 3.1.2: *N*_Pre_ = 89, *N*_Post_ = 104).

**Paraphrasing and establishing relationships**: It was found that both groups paraphrased less after the treatment. In the post-test, the AR group also made more, and the simulation group showed fewer relationships between text and symbol. Despite the possibility of paraphrasing in pictures, the translation performance concentrated on text and symbols. Accordingly, an increase in the number of coding translating paraphrasing from text to text was measured from the pre-test to the post-test (>9.6% in both groups). In comparison, the translations from text to symbol and vice versa decreased in both groups. The treatment only slightly stimulated the AR group to establish relationships between the ERs (see Figure 4).

The translations in 3.1.1.1 *from text to text* were predominantly based on technical terms that were paraphrased using textual definitions. They were used mainly in the pre-test for substance and particle level references. In the post-test, although the use of the texts often intended the substance-particle level change, the participants made this less concrete compared with measurement time 1. The analysis of subcategory 3.1.1.2 *from text to symbol* does not provide any significant findings. The texts of the tasks were primarily chosen in both the pre-test and the post-test in both groups to transfer them into sum or particle forms. Structural formulas tended to be used less. Ultimately, the paraphrasing tasks were predominantly aimed at setting up reaction equations, which is why the participants used molecular or ionic formulae more. In contrast, the reference to the submicroscopic or macroscopic level became more apparent when paraphrasing symbols into text. Although the translations were sometimes imprecise due to inconsistent wording, the deliberate use of technical terms such as “molecule” and the inclusion of technical terms such as “protonation” explicitly emphasized the particle level and did not mix it with the substance level. In the post-test, the participants seemed to pay more frequent and conscious attention to the substance level of their texts. Subcategory 3.1.2 *establishing relationships between ERs* underpins the importance of dealing adequately with the representative level.

**Figure 5:**
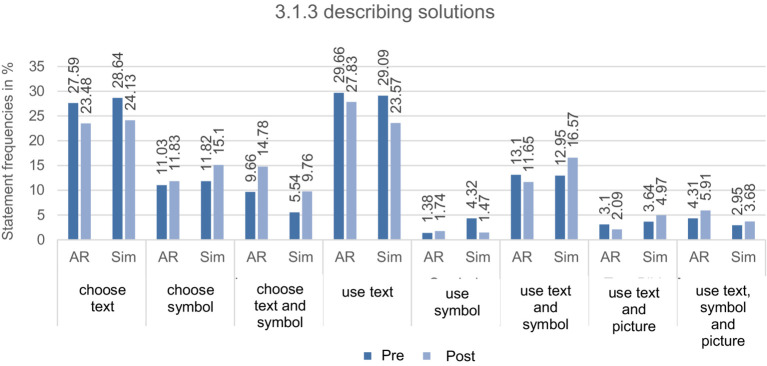
Overview of the relative statement frequencies in percent from CAT of the AR and simulation group (AR and Sim) at measurement times 1 and 2 for the subcategories of 3.1.3 describing solution paths with their total number of statements from pre- and post-test (*N*_Pre_ = 1,020, *N*_Post_ = 1,118).

**Describing solutions**: 3.1.3 *describing solutions* shows a minimal decrease of 0.24% from pre-test to post-test in the AR group and a significant increase of 10.16% in the simulation group about the number of statements. About the choice of (M)ER, the data material shows a similar development of elaboration behavior in both groups. In the post-test, the participants relied less on the text and more on the symbol or its link. When using (M)ER, fewer texts and images with symbols were generally used from the first to the second measurement time point, but minor group differences could be measured. Accordingly, an increase in statements regarding the use of symbols or the combination of three ERs was evident in the AR group. In comparision, the simulation group’s elaboration behavior consistently developed so that the participants used various (M)ERs for their descriptions and primarily resorted to the combination of text and symbol (see Figure 5).

Category 3.1.1.1 demonstrates the difficulties in thinking on the three levels, according to Johnstone (2000). Table 5 shows that subject 3 of the AR group attempted to switch from the particle to the substance level before the treatment but failed to do so due to his lack of language skills. Starting from the description of hydroxide ion deposition and oxidation, the test person wanted to switch to the experimental observation of electroplating. However, more terms are needed at the particle level. In the post-test, the quality of the statements improved. For example, respondent 3 chose this task content again and switched from substance to particle level by not explicitly naming the levels but considering them more differentiated.

**Table 5:** Pre-post comparison with selected anchor examples of respondent 3 of the AR group from subcategory 3.1.3.1 choosing text.

Selected anchor examples from respondent 3 of the AR group	
Pre-test *“That’s where the hydroxide ions go and release electrons. And oxygen, gas and water are produced when the electrons are released. Material and particle level. You can see on one side of the key, […] (which) was previously copper-colored or brass-colored, that it […] is evenly coated with chromium.”* (task 4)	Post-test *“And chromium sulfate sulfate is certainly also somehow colored so that you can see a decrease in color and conclude that the number of chromium ions in solution is decreasing.”* (task 2)

The qualitative content analysis of the respondents’ statements from category 3.1.3.3 *choosing text and symbol* demonstrates that the respondents dealt conscientiously with the substance and particle levels at both measurement times through the combined choice of text and symbol. This result is confirmed by the explicit use of terms such as “atom” or differentiations between the material and particle levels using suitable (M)ERs (e.g., symbols for states of matter to describe the substance level). From category 3.1.3.8 *using text-symbol* about, it becomes clear that using MER often caused issues about thinking in terms of levels, according to Johnstone (2000). Suppose texts and symbols are used simultaneously, for example, by explaining electron transitions with the help of particle formulae. In that case, representation changes are rarely made, technical terms are neglected, and the different ERs of the substance and particle levels are mixed uncontrolled. After the treatment, greater attention was paid to precise technical terminology, and consequently, a more targeted separation of levels was carried out (see Table 6).

**Table 6:** Pre-post comparison with selected anchor examples of respondent 4 of the AR group from subcategory 3.1.3.8 using text and symbol.

Selected anchor examples from respondent 4 of the AR group	
Pre-test *“This means that carbon would be oxidized and lead reduced. So, I set up the partial equations—first, the oxidation equation. Carbon is oxidized to carbon dioxide because of the oxidation number […]. So, I’m now absolutely at the particle level.”* (task 4)	Post-test *“[…] I need H_3_O^+^ ions again and water on the left side […] while the lead ions, which have the oxidation state plus four, then cannot accept four electrons; yes, the lead ions, of course, can accept four electrons and an elementary lead atom would be formed from a lead ion […].”* (task 2)

**Figure 6:**
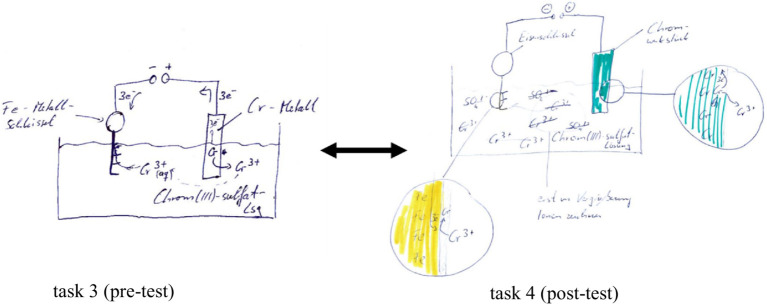
Pre-post comparison of the test processing of respondent 10 of the AR group using the example of the anchor task on electroplating from subcategory 3.1.3.10 describing solutions: using text, symbol, and picture.

The coding of category 3.1.3.10 *using text, symbol, and picture* underpins the trend that the elaboration behavior about dealing with (M)ER in the AR group improved from measurement time 1 to 2 (see below exemplary test processing of the anchor task for galvanization of participant 10):

Optimizations in dealing with (M)ER can be identified, as the participant specifically targeted the substance-particle level exchange by integrating the super magnifier in the experimental sketch (see Figure 6). The respondent conscientiously explained the chemical phenomenon of galvanization by linking the experimental setup of the substance level with the particle processes at the electrodes. The test processing of task 4 of the post-test demonstrates, as an example for the AR group, that applying MER by including the super magnifier causes promising thinking in the levels, according to Johnstone (2000). This effect was not observed in the simulation group.

**Conclusions and diagnoses of errors**: The categorizations of 3.2 on “deep understanding” (Lind et al., 2004) showed a decrease in both groups from the first to the second measurement time point, with the AR and simulation group drawing fewer conclusions in the post-test and making correspondingly more error diagnoses. The breakdown of the findings with (M)ER also showed that both groups used the text less and the symbol or the combination of both ERs more from the pre-test to the post-test. However, the AR setting has resulted in a more significant change in behavior, with significantly less use of text and a much greater focus on MER. Category 3.2.1.1 *text* demonstrates that the subjects of both groups reasoned at a linguistically higher level after processing the respective learning environment. Subject 11, for example, attempted to describe chromium plating at the substance level and explained it at the particle level at the first measurement time. However, he needed to switch appropriately between the levels. In the post-test, he explicitly emphasized in the text that it was necessary to focus on one level (see Table 7).

**Table 7:** Pre-post comparison with selected anchor examples from respondent 11 of the AR group from subcategory 3.2.1.1 reasoning with text.

Selected anchor examples from respondent 11 of the AR group	
Pre-test *“[…] that I have the chromium deposit. In other words, I have the ions beforehand, and then I want it to become a solid,* i.e.*, the atoms.”* (task 3)	Post-test *“Yes, you have to choose one thing again […]. If I start a redox reaction, I must stay at the particle level.”* (task 5)

The behavioral patterns of the two groups when making diagnoses are more distinct from each other and produce a more heterogeneous picture. Although more diagnoses were made with MER in both groups, the AR group focused less on texts and symbols, whereas the simulation group paid less attention to texts and symbols. Subjects who recognized their errors at the representative level also dealt more conscientiously with the substance and particle levels (cf. anchor example selected below, task 5, post-test):

"(I have) mixed up the material and particle levels again. Iron oxide particles to iron atoms. And carbon monoxide molecules to carbon dioxide molecules. That's how I would have to put it." (respondent 11, AR group, subcategory 3.2.3.1 diagnosis of own errors using text).

##### 4.2.1.3 Main category 4: reduction in detailed knowledge through deletion

**Priorities**: The priorities underpin the previous results on dealing with (M)ER in that both groups focused less on texts and more on symbols after processing the respective learning environment. However, their combination was considered more important by the AR group.

**Detail reductions**: From the first to the second measurement time point, texts were considered less important by the AR group and more critical by the simulation group. Texts on material properties such as colors were deleted to evaluate the particle level singularly. Conversely, texts on the particle level, such as “atom,” were deemed unimportant if the substance level was to be emphasized. Accordingly, the AR group did not reduce the details of the symbol in the post-test, although the number of coding in the simulation group increased slightly.

#### 4.2.2 Impact of (immersive) interactivity with the (M)ER of the learning environment

To answer RQ2 (see Chapter 1.1), the data material was analyzed with CAT using, in each case, 10 subjects from the HMD-AR and AR groups (*N* = 20). Given this, 4,175 categorizations were made by both groups. Of these, 2,121 statements can be attributed to the AR group, with 1,106 statements in the pre-test and 1,015 in the post-test. The HMD-AR group made 2,054 statements, of which 1,035 can be attributed to the first measurement time point and 1,019 to the second measurement time point. The percentages in the main categories were almost identical in the two groups at both measurement times. In the post-test, both groups moved slightly less at the level of search-oriented learning and instead elaborated more “understanding-oriented.”

##### 4.2.2.1 Main category 1: adding knowledge elements by retrieving related knowledge from memory

**Mentioning knowledge content and searching for relationships**: The quantitative analysis of category 1.1 *mentioning knowledge elements not dealt with in the task just discussed* demonstrates that the elaboration behavior of the HMD-AR group increased slightly with a percentage share of 1.78%. In contrast, it decreased significantly in the AR group with 7.33%. It was noticeable that the HMD-AR group relied less on knowledge from long-term memory and frequently named knowledge elements from the learning environment. After the treatment, both groups searched for relationships less with the text and more with the symbol, although this development was much more pronounced in the HMD-AR group. Subcategory 1.2.1 *searching for relationships with the text* provides a homogeneous picture. In the pre-test, the search focused more on dealing with the substance and particle levels, whereas in the post-test, questions were generally more related to subject content. The search for relationships with the symbol in the HMD-AR group differs from the AR group in that the focus was less on the triplet relationship (cf. Johnstone, 2000) and more on the particle level. This is demonstrated by the following anchor example from task 4 of the post-test:

"Oh, I've just made a mistake, right? No, that fits. I wasn't sure if I had determined the oxidation number correctly, but that should fit. Exactly, takes up four electrons. This reduces the oxidation number and creates a lead atom." (respondent 45, HMD-AR group, subcategory 1.2.2 search for relationships with the symbol).

##### 4.2.2.2 Main category 3: adding knowledge by generating knowledge through inferences

**Paraphrasing**: If the relative frequencies of subcategory 3.1 *paraphrasing* are considered, similar elaboration trends can be observed in both groups. Paraphrasing from text to text was carried out more frequently in both groups from the first to the second measurement point, with an increase of approximately 9%, whereas translations from text to symbol decreased by approximately 5% in the AR group and approximately 12% in the HMD-AR group. Translations into images were rarely carried out. The AR group translated the symbol into a text significantly less often from the first to the second measurement time point, whereas the HMD-AR group did this more frequently. Overall, paraphrasing into text is particularly important in the HMD-AR group. The qualitative content analysis of category 3.1.1.1 *from the text in the text* provides a similar result in both comparison groups, with the HMD-AR group moving more at the substance level at both measurement times. Their statements are less concerned with particle shapes and their particle processes and more with compound names and their material properties. Substance-particle level changes seem to be disregarded. This gives the impression that the focus of the HMD-AR group, unlike the AR group, was on something other than the triplet relationship (Johnstone, 2000) but on the representative level. The qualitative content analysis of category 3.1.1.4 *from the symbol in the text* demonstrates, analogous to the AR group, a more intensive examination of the substance and particle level. In the post-test, the quality of the statements improved as more precise formulations became apparent and the levels were considered more differentiated.

**Establishing relationships**: This category revealed an increase in coding from pre-test to post-test in both groups, with the AR group establishing more relationships between the ERs with an increase of 3.72% than the HMD-AR group with 1.92%. The post-test showed that after the treatment, the HMD-AR group tried harder to consider the levels independently of each other due to linguistic subtleties. The statements reached a higher linguistic level due to the adequate use of terms from the particle level after processing the HMD-AR learning environment. This is illustrated below with a selected anchor example from task 1 of the post-test:

"Protons are split off from the molecule. This means that the molecular structure changes." (respondent 44, HMD-AR group, subcategory 3.1.2 establishing relationships between ERs).

**Figure 7:**
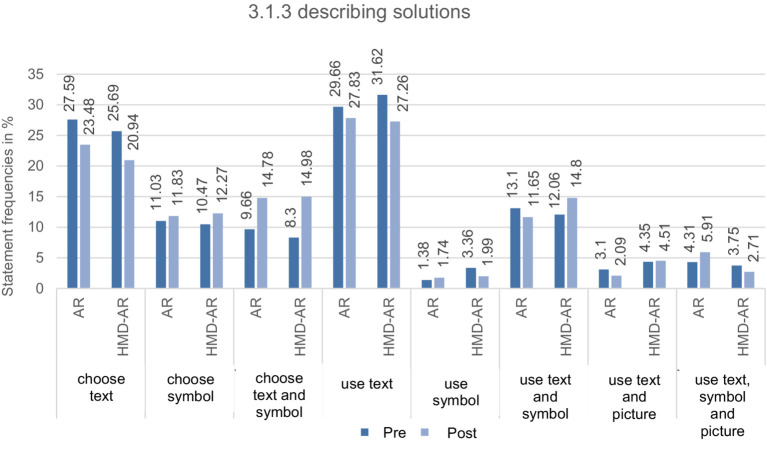
Overview of the relative statement frequencies in percent from CAT of the AR and HMD-AR group (AR and HMD-AR) at measurement times 1 and 2 for the subcategories of 3.1.3 describing solutions with their total number of statements from pre- and post-test (*N*_Pre_ = 1,086, *N*_Post_ = 1,129).

**Describing solution paths**: The quantitative changes from measurement time 1–2 were slightly more significant in the HMD-AR group (3.13%) than in the AR group (0.24%). Figure 7 shows that both groups used the text less and the symbol and MER more, with similar percentage rates of change. This applies in particular to the use of text, whereas the subcategories of 3.1.3 on symbols or MER provide a heterogeneous picture. Accordingly, the HMD-AR group used less the symbol or the combination of all three ERs. However, it relied more heavily on MER in its descriptions, consisting of text, symbols, or images (see Figure 7).

In category 3.1.3.1, as in the AR group, the quality of the statements also increased in the HMD-AR group from pre-test to post-test due to linguistic precision. In the pre-test, statements were elaborated more at the substance or particle level, whereas in the post-test, the changes also played a significant role. The statements from 3.1.3.2 of the HMD-AR group demonstrate that the participants thought primarily at the particle level when choosing symbols. In contrast to the AR group, however, the HMD-AR group only showed a limited improvement in content quality from the pre-test to the post-test. Although both groups were more conscientious of particle-level terms such as “atom” in the post-test, the HMD-AR group often failed to consider the change from substance to particle level (and vice versa).

**Figure 8:**
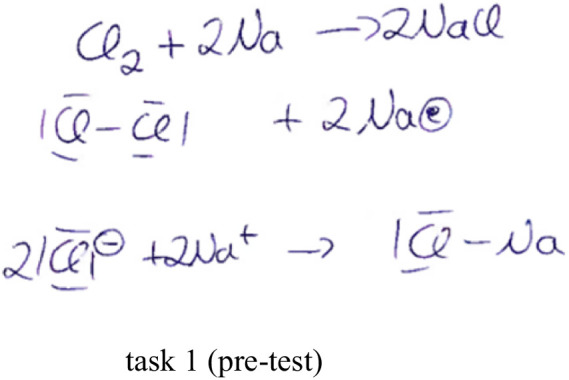
Test processing of respondent 46 of the HMD-AR group using the example of task 1 of the pre-test from subcategory 3.1.3.8 describing solutions: using text and symbols.

Subcategory 3.1.3.8 *text and symbol*, which can be predominantly assigned to the particle level, also illustrates the difficulties in dealing specifically within a level. Figure 8 shows this using the test processing of participant 46 of the HMD-AR group as an example. Although his notations and verbal utterances were strictly at the particle level, he had problems with the correct symbol representation of the salt particle, “sodium chloride.” He incorrectly used the valence line notation instead of sketching the crystal structure in the particle structure. The notation is also of poor quality about the number of particles, as the coefficients were neglected in the last step.

Furthermore, numerous codes in this category increasingly integrate material properties into the statements at the particle level. The results confirm that the HMD-AR group, unlike the AR group, intended fewer substance-particle level changes and tended to try to differentiate between the two levels. The data material indicates improved elaboration behavior in both groups from the first to the second measurement time point. Whereas the AR group tended to carry out more determined substance-particle level changes in the post-test, the HMD-AR group elaborates more conscientiously at the individual level, especially at the particle level, using text and symbols (cf. Figure 8).

**Figure 9:**
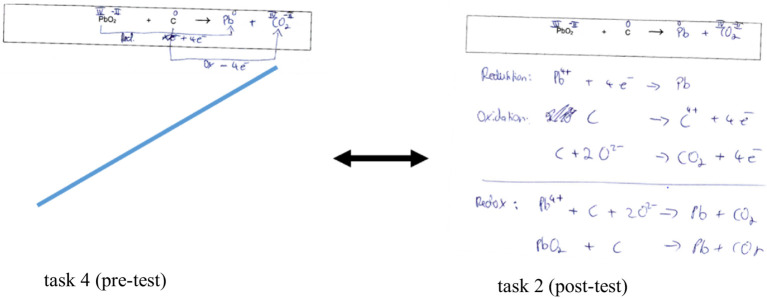
Pre-post comparison of the test processing of respondent 43 of the HMD-AR group using the example of the anchor task for extracting lead from subcategory 3.1.3.8 describing solutions: using text and symbol.

**Figure 10:**
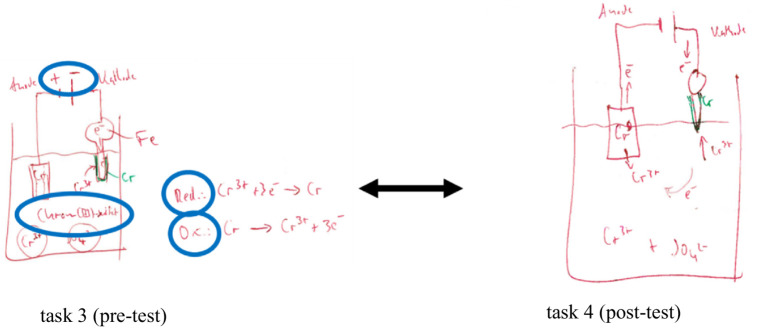
Pre-post comparison of respondent 52’s test processing using the example of the anchor task on electroplating from subcategory 3.1.3.10 describing solutions: using text, symbol, and picture.

Figure 9 illustrates that subject 43 only hints at the electron transitions at the first measurement time and verbalizes the associated processes. In contrast, the written descriptions in the post-test already indicate a more conscientious examination of the particle level. After the treatment, the HMD-AR group dealt more carefully with the representative and submicroscopic levels. In contrast, the AR group thought more in all three levels, according to Johnstone (2000). Category 3.1.3.10 *using text, symbol, and image* confirms this. However, this effect was not diagnosed in the HMD-AR group. The HMD-AR group moved erratically between the levels and neglected concrete explanations of the substance-particle level change. The anchor example of test subject 52 (see Figure 10) demonstrates that no changes were registered in the HMD-AR group from pre-test to post-test. The participant combined text, symbol, and image without separating the substance and particle levels (e.g., image without separating the levels with a super magnifying glass).

**Conclusions and diagnoses of errors**: The coding to “deep understanding” (Lind et al., 2004) decreased in both groups. To an almost identical extent, with a percentage difference of approximately 11% of the participants inferred less using the given (M)ER and diagnosed their errors more frequently. Both comparison groups used fewer texts and more symbols to draw conclusions and analyze their errors. Furthermore, the combination of text and symbol was increasingly used for diagnoses. Conclusions using MER were only made more frequently in the AR group in the post-test. The qualitative content analysis confirms an improvement in elaboration behavior from pre-test to post-test in both groups to thinking on the three levels, according to Johnstone (2000). Once again, it is clear that the HMD-AR group sought to change substance-particle levels to a lesser extent.

The anchor example of respondent 41, as shown in Table 8, demonstrates that in the pre-test, the text was used to conclude at the particle level. However, the level needed to be clearly described and explained. Therefore, it is unclear which particle increases its oxidation state and whether the respondent consciously focused on the particle level. If a statement from the post-test is examined, the linguistic precision using technical terms such as “atoms” and “ions” becomes apparent. Accordingly, he set the meaning of the representative level to the particle level.

**Table 8:** Pre-post comparison with selected anchor examples of respondent 41 of the HMD-AR group from subcategory 3.2.1.1 reasoning with text.

Selected anchor examples from respondent 41 of the HMD-AR group	
Pre-test *“This means that increases the oxidation state increases when the electrons are released.”* (task 3)	Post-test *“Because they are not beads. If they were, you’d have to say, and so on whether they were atoms, ions, molecules,* etc.*”* (task 5)

##### 4.2.2.3 Main category 4: reduction in detailed knowledge through deletion

**Setting priorities and reducing details**: It turned out that from the first to the second measurement time point, the participants in both groups placed more emphasis (approximately 12%) and reduced details (>13.9%). This underpins the previous findings that the text tended to move into the background and the symbols and MER into the center of the elaboration. The qualitative content analysis of categories 4.2.1.1 e*mphasis on the symbol* and 4.2.1.3 *emphasis on text and symbol* again revealed that the HMD-AR group dealt more intensively with the representative level in the post-test. In addition, the HMD-AR group described their approach to ER selection and application more clearly in the post-test than in the pre-test. This is exemplified by a statement from respondent 43, who concentrated on the uniform expression by combining particle shape and technical term to describe the reduction (see selected anchor example below, task 5, post-test):

"Material language mixed with symbolic language, so to speak. So there are different names. I would rather say uniformly: reduction of copper ions. And then maybe also say: Cu2^+^ and the oxidation of solid iron, Fe or solid iron atom even, solid iron is better, elemental copper, Cu, and iron oxide FeO, could be done." (respondent 43, HMD-AR group, subcategory 4.2.1.3 emphasis on text and symbol).

This conscious confrontation with the (M)ER was diagnosed less frequently in the statements of the AR group.

#### 4.2.3 Conclusion of the results

The quantitative frequency and qualitative content analysis of the categorizations revealed that the conceptual preparation of the non- and AR learning environment had encouraged the test subjects to engage more deeply with the (M)ER at substance and particle levels. The trend became apparent that working with the AR learning environment, in particular, resulted in more intensive elaboration at the substance level and that the teachers in the AR group elaborated more in an understanding-oriented and less in a search-oriented manner from measurement time 1 to 2. In the pre-test, difficulties were diagnosed in both groups about dealing with the substance and particle level, which were often based on a lack of language skills (neglect of technical terms or mixing of (M)ER). In addition, switching levels was a significant challenge. After the treatment, all subjects established more relationships between the levels and switched between them more decisively. In addition, from pre-test to post-test, the text moved into the background, so symbols and MER tended to be weighted more. In the post-test, the teachers used (M)ER more systematically to explain the substance and particle level, which favored successful level changes. Overall, the results indicate that the AR setting had a more significant effect on cognitive processing (in the sense of a more deciduous handling of the three levels according to Johnstone, 2000) when using (M)ER at the substance and particle levels. The AR group differed from the simulation group in that they were visibly more conscientious with the representative level after the treatment differentiated more successfully between the substance and particle levels. The results also show that working with the “classic” AR setting on the tablet resulted in more significant behavioral changes concerning the understanding of the substance-particle concept than on the AR glasses. The test subjects who worked with AR in tablet format achieved more positive effects about their understanding of the substance-particle concept than with the HMD-AR technique: the AR group distinguished itself from the HMD-AR group through its more conscientious handling of the substance level and the associated more adequate level change. Overall, it became apparent after the treatment that the focus was on the adequate handling of (M)ER about the targeted integration of technical terms (e.g., “anions”) and the explication of the substance and particle level. Analogous to the AR group, the text as a form of representation receded into the background from measurement time 1 to 2, whereas symbols and MER gained importance. One particular result was that the AR group tried to think more on all three levels, whereas the HMD-AR group tended to concentrate more on the representative level. Accordingly, the analyses provide initial indications that the AR learning environment on the AR glasses tends to positively stimulate chemical terminology and less the understanding of substance-particle concepts (cf. Figure 10 and Table 8).

## 5 Discussion

**RQ1** examines whether the AR learning environment can be applied to promote the use of chemical terminology among chemistry teachers (cf. Chapter 1.1). To assess the effects of AR on the use of (M)ER, the statements from the test responses of the simulation and AR group, evaluated with CAT, were examined. From a qualitative perspective, both comparison groups elaborated the pre-test extent similarly. In line with the findings of the international literature, the evaluations demonstrated that the teachers had immense problems dealing adequately with the representative level (Treagust et al., 2003; Erlenbach and Frank, 2022). The “search-oriented learning” (Schmalhofer, 1996) was based on a knowledge recall with terms and short definitions at the first measurement time. In contrast, more detailed explanations and descriptions of the subject content were retrieved in both groups in the post-test. The search for relationships in the pre-test was also characterized by imprecise formulations in both groups, which incorrectly “mixed” the substance level with the particle level. This elaboration behavior changed in both groups from the first to the second measurement time. The initial difficulties in dealing with the chemical terminology of the pre-test were visibly reduced in the post-test, as the participants, especially in the AR group, articulated more consistently with the ER and separated texts from symbols more conscientiously. In the pre-test, text and symbol were often used simultaneously and thus disturbed the uniform expression. This was because technical terms were neglected, which led to the nebulous mixing of substance and particle levels. Such statements were diagnosed less frequently in the post-test. The importance of symbols increased immensely from the first to the second measurement time points. It led to higher quality statements in both groups during superficial learning and particularly in the AR group during deep learning. The results demonstrate that when the terms “atom,” “ion,” and “molecule” were taken into account, level changes were carried out more decisively. The evaluation showed that participants who recognized their errors at the representative level also dealt more conscientiously with the substance and particle levels. Accordingly, different elaboration profiles were revealed in both comparison groups from pre-test to post-test, which indicates improved handling of chemical terminology. The “understanding-oriented learning” shifted from the focus on the (M)ER of the particle level to the substance level (e.g., by naming definitions for level explication). Concentrating on the substance level using the (M)ER often led to clear level separations and changes. In the post-test, the cognitive schemata appear to have been more consciously linked to the thought processes in working memory. AR appears to have initiated cognitive processing based on symbols and MER, which should have favored the development of a textbase and a mental image (cf. Habig, 2019; Altmeyer et al., 2020). The teachers’ domain-specific previous knowledge (Sweller et al., 1998; Kroß and Lind, 2001; Chi, 2006; Tricot and Sweller, 2014) should have been activated by the treatment to support the construction of mental models. On the one hand, it is possible that the cognitive schemata with strong references to the substance-particle level change were already present before the treatment (Schnotz, 2001b). However, they could not be recalled at measurement time one and were only activated by the more conscientious handling of the substance level during the treatment. On the other hand, it is conceivable that the treatment stimulated thinking in the three levels (Johnstone, 2000), to construct mental models to the level changes while working with the learning environment, which was then transferred to long-term memory and recalled at measurement time 2 (Johnson-Laird et al., 2018). They could also have been constructed directly during the elaboration of the post-test. Although the test responses at the second measurement time point cannot provide any information about the timing of the model constructions for dealing with (M)ER, it is assumed that previous knowledge played a central role and had a dominant influence on the mental image or text base (Kroß and Lind, 2001; Schnotz, 2001b). The elaboration behavior of both groups at measurement time 1 was more similar to that of novices with low previous knowledge, as they dealt with the texts for longer (Schnotz, 2014) and at measurement time 2 to that of experts with higher previous knowledge, because they elaborated the symbols visibly longer and more thoroughly (Zhao et al., 2020). The AR group showed greater concentration and a more conscientious approach to the representative level than the simulation group. Whereas the simulation group described solutions heterogeneously with the variety of all (M)ERs, the AR group tended to focus more on combining text, symbol, and picture. Above all, this combination of ER seems to have strengthened the adequate handling of the three levels according to Johnstone (2000), especially when the tool “Super-magnifying glass” was integrated into the sketches of the real experiment. The emphasis on the super magnifier was characteristic of successful elaboration behavior, but this was only evident in the AR group. This result indicates that since the AR setting avoids split-attention (Ayres and Sweller, 2021) and links the virtual particle processes spatially and temporally with the material level (cf. contiguity principle according to Fiorella and Mayer, 2021), cognitive modeling processes were initiated and reconstructed in the test processing at measurement time 2. Accordingly, the data material provides the trend that AR optimized the use of chemical terminology and that the understanding of the substance and particle level (Talanquer, 2011) developed further. This finding can be reconciled with the results by Radu and Schneider (2019), who found that AR can positively affect multimedia learning (Mayer, 2014; Schnotz, 2005). Based on the study results by Thees et al. (2020) and Buchner and Zumbach (2020), AR should provide promising support for learning processes. The explanations above confirm the hypothesis that using the AR learning environment supports using chemical terminology in a learning-effective manner. This finding is congruent with the results of the physics didactics experts Altmeyer et al. (2020), who investigated the effects of (non-)AR-supported learning settings in the physics laboratory on conceptual knowledge in connection with the cognitive load theory (Chandler and Sweller, 1991). Their comparative study revealed that both the non-AR and AR-supported learning environments, both accessible via tablet, had a positive influence on real-life experimentation. No clear group differences could be diagnosed in the present study either. Only trends indicate that a tendency toward developing different elaboration profiles in the two groups through AR forms of representation in real experiments were identified (Altmeyer et al., 2020). A significant increase in learning in the AR group was not measured directly. Therefore, it can be summarized that the AR innovation’s theoretically derived and assumed potential could not be fully confirmed. Although contiguity could not be ensured in the “classic” digital learning environment without AR, a split-attention effect was, in all probability, not measured directly (Altmeyer et al., 2020). Since it was found that the simulation-based learning environment also has a supportive effect on the handling of (M)ER, neither split-attention nor the lack of contiguity seems to have a noticeable influence on the cognitive resources of the teachers (Sweller, 2011; Mayer, 2014; Fiorella and Mayer, 2021). This finding is of great significance, as the split-attention effect was originally estimated to be significant in real experiments in the laboratory. Consequently, AR and non-AR differ only slightly from each other. This suggests similar cognitive information processing in working memory (see Schnotz, 2011; Altmeyer et al., 2020; Thees et al., 2020). Hypothesis H1 (cf. Chapter 1.1) can, therefore, only be confirmed to a limited extent.

**RQ2** investigates the extent to which the interactive use of (immersive) AR forms of representation in the learning environment can positively influence the use of chemical terminology and whether different elaboration profiles develop depending on the medium (see Chapter 1.1). To investigate the effect of interactivity with AR on the use of (M)ER, the statements from the test responses of the AR and HMD-AR groups from measurement times 1 and 2, evaluated with CAT, were analyzed quantitatively and qualitatively. If the coding of the pre-test is examined more closely, the difficulties in dealing with chemical terminology and, finally, the understanding of substance-particle concepts were also found in the HMD-AR group. Consequently, these results support the fact that a lack of language skills (e.g., arbitrary linking of (M)ERs) has a negative influence on the switch from substance to particle level (and vice versa). Both comparison groups had similar difficulties dealing with the representative level in the pre-test (cf. Treagust et al., 2003; Erlenbach and Frank, 2022). An interesting aspect of elaborating the pre-test was that “ion” was often used for charged particles, but atoms in their ground state were not referred to as such. This example describes the inconsistent linguistic expressions and points to the need to differentiate between all particles, charged and uncharged. It also showed that the statements on the substance level were mostly aimed at compound names and rarely at substance properties such as color or deformability. However, focusing on material properties is important for thinking on the three levels (Johnstone, 2000). The qualitative group comparison from the pre-and post-test revealed a more conscious engagement with chemical terminology in both groups. Whereas a more conscientious engagement with the substance level was measured in the AR group, the HMD-AR group only provided initial indications. The statements of both groups in the post-test were based less on texts and more on symbols and their combinations. In this context, detailed sketches and symbol spellings were described more. One difficulty for teachers is explaining processes at the particle level using symbols. The increased use of symbols in the post-test suggests improved elaboration behavior, as teachers are more likely to understand the importance of models at the submicroscopic level (Farida et al., 2010). Therefore, it is reasonable to suspect that AR promotes conscious engagement with symbols, whereby the mental model enriched with knowledge from long-term memory was stimulated in working memory and thinking in the three levels, according to Johnstone (2000). The HMD-AR group was more conscientious in using technical terms, making more references between text and symbol about the substance or particle level but rarely seeking to switch levels. In the HMD-AR group, successful substance-particle level changes in the form of mentions of the super magnifying glass were sometimes diagnosed due to the use of (M)ER, but this was rarely the case. Beyond that, the evaluation demonstrates that intended level changes of the HMD-AR group were often even incorrect in the post-test. Although both groups appear to have elaborated more on the level of “deep understanding” (cf. Schmalhofer, 1996) in the post-test, the more conscientious handling of (M)ER at the substance level as a positive effect was especially evident in the HMD-AR group. The AR group measured the visibly improved understanding of substance-particle concepts in particular. Whereas the AR group often focused explicitly on the particle model, the HMD-AR group dealt with the (M)ER *per se*. A sign of the more conscientious handling of the representative level through immersive AR objects could be using (M)ER without integrating the substance and particle level. Teachers only focus on the representative level if they cannot develop a suitable solution through the relationship between the substance, particle, and representative levels. Consequently, in the HMD-AR group, using (M)ER seems to replace elaboration at the substance *and* particle levels. Instead of the links between the ERs were considered, individual forms of representation were described, and explanations were made at most about the substance *or* particle level. When the two levels were considered specifically, the behavior and thought patterns from the pre- and post-test confirmed that an appropriate level of technical language focusing on coefficients or technical terms such as “ions” could result in a more purposeful approach to the substance and particle level. For example, the results of the HMD-AR group revealed that the use of text and symbols led to more conscientious elaboration at the particle level and a more planned approach to the tasks. Due to the linguistic subtleties, such as the adequate use of terms at the particle level, the statements of the HMD-AR group then, consciously or unconsciously, attained a higher linguistic level. However, the qualitative analyses did not indicate an improved understanding of substance-particle concepts after working with the AR glasses. Although ensuring contiguity and avoiding split-attention when using the HMD-AR technique should have positive effects (see Chapter 1.1), a high extraneous load could have led to cognitive overload (Sweller, 2011). This could explain why HMD-AR hinders thinking in the three levels (Johnstone, 2000) but promotes concentration on (M)ER itself. This suspicion is in line with the findings by Buchner et al. (2021), who rate the learning-promoting potential of AR glasses as low. If the immersive experience completely merges the substance and particle levels, a learning-related cognitive load could be very obvious (Chandler and Sweller, 1991). AR on the tablet tends to refer more strongly to the model character due to the framed view on the screen. Accordingly, the digital medium concretely separates the real experimental setup at the material level from the particle processes of the learning environment. To a certain extent, the tablet acts as a “barrier” between the levels and presumably prevents the continuum view. Although the contents of both learning environments refer to particle modeling, the work with the HMD-AR technology gave the impression that this information could not always be accessed. In some cases, the participants seem to have had problems operating the AR glasses despite extensive instruction in their technical handling. Furthermore, the wearing comfort of the glasses left something to be desired (Scheerer, 2021; Kaufeld et al., 2022). It may have been too unfamiliar for the participants to click on immersive AR objects. If the instructions are difficult to carry out due to technical challenges, extremely negative effects are likely to be evoked from a motivational perspective. Furthermore, in contrast to the classic AR variant on the tablet, the immersive experience could provoke the idea of a continuum (Staatsinstitut für Schulqualität und Bildungsforschung, 2023b). The boundary between the virtual objects as particles and the real objects at the substance level is no longer recognizable and disrupts cognitive processing. If the augmented (M)ER visualization on the glasses does not adequately differentiate the levels from one another, this could lead to faulty modeling processes (Schnotz and Bannert, 2003; Staatsinstitut für Schulqualität und Bildungsforschung, 2023b). This would result in constructing incorrect mental models that could not be linked to the existing previous knowledge from long-term memory (Schnotz, 2001a). One explanation for this is cognitive overload, which is based on the intrinsic and extraneous load types (Peeters et al., 2023). If the participant does not have sufficient cognitive resources during the elaboration with the HMD-AR technology (Chandler and Sweller, 1991), an overload may follow (Buchner et al., 2021; Peeters et al., 2023). If an attempt was then made to recall an erroneous mental image or test basis at measurement time 2, the successful surface and deep elicitation were not only disturbed but negatively manipulated (Johnson-Laird et al., 2018). This could explain why the HMD-AR group could not link all three levels, according to Johnstone (2000). Since handling the representative level in the HMD-AR group has been based on far more intensive modeling processes than in the AR group, hypothesis H2 tends to be confirmed. The trend revealed that AR in its classic tablet format could be conducive to learning and understanding the substance-particle concept and, thus, implicitly for dealing with (M)ER. Above all, the immersion experience with the AR glasses seems to have a positive effect on the use of chemical terminology. Thinking in the three levels (Johnstone, 2000) was at best implicitly positively or even negatively influenced, presumably due to the frequently diagnosed challenges in dealing with the AR glasses (Kapici, 2023). As a result, two elaboration profiles seem to have developed, depending on the interactive use of the AR representations and their immersion strength.

**Conclusion**: As described in Chapter 1, both studies confirm that the teachers consistently had immense problems at measurement time 1 in dealing adequately with chemical terminology. This was characterized by imprecise formulations, which led to various mix-ups of the levels, according to Johnstone (2000). Main study 1 revealed that the conceptual preparation of the learning environments, AR and non-AR, changed the elaboration behavior from the first to the second measurement point, as the difficulties in dealing with the representative level were visibly reduced. In particular, the importance of symbols increased. It is also concluded that concentrating on the representative level leads to a more conscientious approach to the substance and particle levels. The research project reveals that a digital learning environment’s media and subject-specific didactic design, mainly through integrating suitable symbols and MER, can positively enrich cognitive processing. AR has great potential in promoting (M)ER use and, as a result, the understanding of substance-particle concepts. Avoiding split-attention and the spatial and temporal linking of substance and particle levels can support the initiation of modeling processes. However, as the differences between AR and non-AR were moderate, a split-attention effect could not be measured directly. The learning effectiveness of AR appears to be present in the use of (M)ER but not significantly more pronounced than in the simulation-based learning environment. Main study 2 revealed that not only AR but also, in particular, HMD-AR led to a more conscious engagement with the representative level. Here, HMD-AR technology seems to favor using symbols and MER. Although the HMD-AR group made more references between the representations on the substance and particle level and dealt more conscientiously with chemical terminology *per se*, according to Johnstone (2000), thinking on all three levels appears to have been only moderately improved by the treatment. After processing the HMD-AR learning environment, unlike the AR group, hardly any more determined substance-particle level changes were made. It is reasonable to assume that, despite the positive results of dealing with (M)ER, the HMD-AR technique led to cognitive overload. Therefore, AR in its classic tablet form is beneficial for learning and understanding the substance-particle concept and, thus, implicitly also for dealing with (M)ER. In comparison, the immersion experience on the AR glasses positively affects the use of chemical terminology and less on thinking in the three levels (Johnstone, 2000).

## Data availability statement

The raw data supporting the conclusions of this article will be made available by the authors, upon reasonable request.

## Ethics statement

The studies involving humans were approved by the ethics Committee of the Technical University of Munich (cf. Volume 1). The studies were conducted in accordance with the local legislation and institutional requirements. The participants provided their written informed consent to participate in this study.

## Author contributions

MR: Writing – original draft, Writing – review & editing. CN: Writing – original draft, Writing – review & editing.

## Footnotes

^1^The anchor example, as well as all subsequent examples, has been translated from German into English.

## References

[ref1] Ainsworth S. E. (1999). The functions of multiple representations. Comput. Educ. 33, 131–152. doi: 10.1016/S0360-1315(99)00029-9

[ref2] Akçayır M. Akçayır G. (2017). Advantages and challenges associated with augmented reality for education: a systematic review of the literature. Educ. Res. Rev. 20, 1–11. doi: 10.1016/j.edurev.2016.11.002

[ref3] Al-Balushi S. (2012). The effect of macroscopic and submicroscopic pictorial representations on pre-service science teachers’ explanations. Int. J. Acad. Res. 4, 10–14. doi: 10.7813/2075-4124.2012/4-6/B.2

[ref4] Altmeyer K. Kapp S. Thees M. Malone S. Kuhn J. Brünken R. (2020). The use of augmented reality to foster conceptual knowledge acquisition in STEM laboratory courses—theoretical background and empirical results. Br. J. Educ. Technol. 51, 611–628. doi: 10.1111/bjet.12900

[ref5] Anderson M. Anderson S. L. (2019). How should AI be developed, validated, and Imple mented in patient care? AMA J. Ethics 21, E125–E130. doi: 10.1001/amajethics.2019.125 30794121

[ref6] Ayres P. Sweller J. (2021). “The Split-attention principle in multimedia learning” in The Cambridge handbook of multimedia learning. eds. Mayer R. E. Fiorella L. (Cambridge, United Kingdom: Cambridge University Press), 199–211.

[ref7] Azuma R. T. (1997). A survey of augmented reality. Presence Teleop. Virt. 6, 355–385. doi: 10.1162/pres.1997.6.4.355

[ref8] Bacca J. Baldiris S. Fabregat R. Graf S. (2014). Augmented reality trends in education: a systematic review of research and applications. J. Educ. Technol. Soc. 17, 133–149. doi: 10.2307/jeductechsoci.17.4.133

[ref9] Bannert M. (2009). Promoting self-regulated learning through prompts. Zeitschrift für Pädagogische Psychologie 23, 139–145. doi: 10.1024/1010-0652.23.2.139

[ref10] Bortz J. Döring N. (2006). Forschungsmethoden und Evaluation für Human-und Sozialwissenschaftler. 4th Edn. Berlin-Heidelberg: Springer-Verlag.

[ref11] Buchner J. Buntins K. Kerres M. (2021). A systematic map of research characteristics in studies on augmented reality and cognitive load. Comput. Educ. Open 2, 100036–100038. doi: 10.1016/j.caeo.2021.100036

[ref12] Buchner J. Freisleben-Teutscher C. (2020). Die Erweiterung der Realität als Bildungschance: Fallbeispiele für immersives Lernen in Schule und Hochschule. In Beinsteiner A. Blasch L. Hug T. Missomelius P. Rizzoli M. (Hrsg.), Medien-Wissen-Bildung. Augmentierte und virtuelle Wirklichkeiten (1. Aufl., S. 175–188). Innsbruck, Austria: Innsbruck University Press.

[ref13] Buchner J. Zumbach J. (2020). Augmented reality in teacher education: a framework to support teachers’ technological pedagogical content knowledge. Italian J. Educ. Technol. 28, 106–120. doi: 10.17471/2499-4324/1140

[ref14] Bürg O. (2005). “Akzeptanz von E-Learning in Unternehmen” in Die Bedeutung von institutionellen Rahmenbedingungen, Merkmalen des Individuums und Merkmalen der Lernumgebung für die Akzeptanz von E-Learning, vol. 4 (Berlin: Logos Verlag), 75–85.

[ref15] Chandler P. Sweller J. (1991). Cognitive load theory and the format of instruction. Cogn. Instr. 8, 293–332. doi: 10.1207/s1532690xci0804_2

[ref16] Chavan S. R. (2016). Augmented reality vs. virtual reality: differences and similarities. Int. J. Adv. Res. Comput. Eng. Technol. 5, 1–6. doi: 10.7326/M13-0830

[ref17] Chi M. T. H. (2006). “Two approaches to the study of experts' characteristics” in The Cambridge handbook of expertise and expert performance. ed. Ericsson K. A. (Cambridge, United Kingdom: Cambridge University Press).

[ref18] Crawford B. A. Cullin M. J. (2004). Supporting prospective teachers' conceptions of modelling in science. Int. J. Sci. Educ. 26, 1379–1401. doi: 10.1080/09500690410001673775

[ref19] Davis F. D. (1989). Perceived usefulness, perceived ease of use, and user acceptance of information technology. MIS Q. 13, 319–340. doi: 10.2307/249008

[ref20] Derman A. Ebenezer J. (2020). The effect of multiple representations of physical and chemical changes on the development of primary pre-service teachers cognitive structures. Res. Sci. Educ. 50, 1575–1601. doi: 10.1007/s11165-018-9744-5

[ref21] Devetak I. Urbančič M. Wissiak Grm K. S. Krnel D. Glažar S. A. (2004). Submicroscopic representations as a tool for evaluating students' conceptions. Acta Chim. Slov. 51, 799–814. doi: 10.1002/9780470344594.ch3

[ref22] Döring N. Bortz J. (2016). Forschungsmethoden und Evaluation in den Sozial-und Humanwissenschaften. 5. Auflage Edn. Berlin Heidelberg: Springer.

[ref23] Dunleavy M. Dede C. (2014). “Augmented reality teaching and learning” in Handbook of research on educational communications and technology. eds. Spector M. J. Merrill D. M. Elen J. Bishop M. J. (New York: Springer), 735–745.

[ref24] Eilks I. (2012). The role and potential dangers of visualisation when learning about sub-microscopic explanations in chemistry education. Centre for Educational Policy Studies Journal, 125–145. doi: 10.26529/cepsj.322

[ref25] Ericsson K. A. Simon H. A. (1993). Protocol analysis: verbal reports as data. Rev. Edn. Cambridge, MA: Bradford Books/MIT Press.

[ref26] Erlenbach R. Frank C. (2022). Rolle des Vorwissens beim Lernen mit externalen Repräsentationen: Theoriegeleitete Ableitung und systematisches Literaturreview. Unterrichtswissenschaft 50, 479–516. doi: 10.1007/s42010-022-00143-0

[ref27] Farida I. Widyantoro D. H. Sopandi W. (2010). Representational competence’s profile of pre-service chemistry teachers in chemical problem solving: International Seminar on Science Education. Bandung, Indonesia.

[ref28] Figl K. (2010). Deutschsprachige Fragebögen zur Usability-Evaluation im Vergleich. Zeitschrift für Arbeitswissenschaft 4, 321–337. doi: 10.1007/s41449-019-00060-1

[ref29] Fiorella L. Mayer R. E. (2021). “Principles for reducing extraneous processing in multimedia learning” in The Cambridge handbook of multimedia learning. eds. Mayer R. E. Fiorella L. (Cambridge, United Kingdom: Cambridge University Press), 185–198.

[ref30] Fleischer T. Tatzgern M. Deibl I. Zumbach J. (2022). “Das virtual reality Chemielabor Chem GerLab–Experimentieren in der virtuellen Realität” in Edition Fachdidaktiken. Digitale NAWIgation von Inklusion: Digitale Werkzeuge für einen inklusiven Naturwissenschaftsunterricht. eds. Watts E. M. Mann C. H. (Wiesbaden, Germany: Springer Fachmedien Wiesbaden), 115–122.

[ref31] Garzón J. Acevedo J. (2019). Meta-analysis of the impact of augmented reality on stu dents’ learning gains. Educ. Res. Rev. 27, 244–260. doi: 10.1016/j.edurev.2019.04.001

[ref32] Goldkuhle P. (1993). Modellbildung und Simulation im Physikunterricht. Soest: Verlagskontor.

[ref33] Goodhue D. L. (1995). Understanding user evaluations of information systems. INFORMS 41, 1827–1844. doi: 10.1287/mnsc.41.12.1827

[ref34] Guthke T. Beyer R. (1992). Inferenzen beim Satz-und Textverstehen. Z. Psychol. 200, 321–344.

[ref35] Habig S. (2019). Augmented reality chemistry–Förderung internaler Modellrepräsentation in Organischer Chemie durch AR. Bonn, Germany: Gesellschaft für Informatik e.V.

[ref36] Harrison A. G. Treagust D. F. (2000). A typology of school science models. Int. J. Sci. Educ. 22, 1011–1026. doi: 10.1080/095006900416884

[ref37] Hellriegel J. Čubela D. (2018). Das Potenzial von Virtual Reality für den schulischen Unterricht-Eine konstruktivistische Sicht. Medien Pädagogik: Zeitschrift für Theorie und Praxis der Medienbildung, 58–80.

[ref38] Hubermann M. (1991). Der berufliche Lebenszyklus von Lehrern: Ergebnisse einer empirischen Untersuchung, Köln (Cologne), Germany: Böhlau Verlag 249–267.

[ref39] Huwer J. Lauer L. Dörrenbächer-Ulrich L. Perels F. Thyssen C. (2019). Chemie neu erleben mit Augmented Reality: Neue Möglichkeiten der individuellen Förderung. MNU J, 72, 420–427. doi: 10.1016/j.chb.2019.06.020

[ref40] Ibanez M.-B. Delgado-Kloos C. (2018). Augmented reality for STEM learning: a systematic review. Comput. Educ. 123, 109–123. doi: 10.1016/j.compedu.2018.05.002

[ref42] Johnson-Laird P. N. Goodwin G. P. Khemlani S. S. (2018). “Mental models and reasoning” in The Routledge international handbook of thinking and reasoning. eds. Ball L. J. Thompson V. A., London, United Kingdom: Routledge 346–365.

[ref43] Johnstone A. H. (1993). The development of chemistry teaching: a changing response to changing demand. J. Chem. Educ. 70, 701–705. doi: 10.1021/ed070p701

[ref44] Johnstone A. H. (2000). Teaching of chemistry – logical or psychological? Chem. Educ. Res. Pract. Eur. 1, 9–15. doi: 10.1039/A9RP90001B

[ref45] Jonkisz E. Moosbrugger H. Brandt H. (2012). “Planung und Entwicklung von Tests und Fragebogen” in Springer-Lehrbuch. Testtheorie und Fragebogenkonstruktion. eds. Moosbrugger H. Kelava A.. 2. Aufl., S ed (Berlin Heidelberg: Springer), 27–74.

[ref46] Justi R. S. Gilbert J. K. (2002). Models and modelling in chemical education. In: Gilber J.G. Jong O. De Justi R. Treagust T.F. Driel J.H. Van (Hrsg.). Chemical education: Towards research based practice. Dordrecht, Niederlande: Kluwer, 47–68.

[ref47] Kalyuga S. Sweller J. (2014). “The redundancy principle in multimedia learning” in The Cambridge handbook of multimedia learning. ed. Mayer R. E.. 2nd (Cambridge, United Kingdom: Cambridge University Press), 247–262.

[ref48] Kapici H. O. (2023). From symbolic representation to submicroscopic one: preservice science teachers’ struggle with chemical representation levels in chemistry. Int. J. Res. Educ. Sci. 9, 134–147. doi: 10.46328/ijres.3122

[ref49] Kaufeld M. Mundt M. Forst S. Hecht H. (2022). Optical see-through augmented reality can induce severe motion sickness. Displays 74:102283. doi: 10.1016/j.displa.2022.102283

[ref50] Keller S. Habig S. (2022). “Supporting spatial thinking in organic chemistry through augmented reality—an explorative interview study” in Student reasoning in organic chemistry. eds. Graulich N. Shultz G. (Cambridge, United Kingdom: The Royal Society of Chemistry), 19–35.

[ref51] Kintsch W. (1993). Information accretion and reduction in text processing: inferences. Discourse Process. 16, 193–202. doi: 10.1080/01638539309544837

[ref52] Klos S. Henke C. Kieren C. Walpuski M. Sumfleth E. (2008). Naturwissenschaftliches Experimentieren und chemisches Fachwissen - zwei verschiedene Kompetenzen. Zeitschrift für Pädagogik 54, 304–321. doi: 10.1002/pad.537

[ref53] Kopp B. Dvorak S. Mandl H. (2003). Evaluation des Einsatzes von Neuen Medien im Projekt "Geoinformation-Neue Medien für die Einführung eines neuen Querschnittfachs". Pädagogische Psychologie, (Forschungsbericht Nr. 161). Available at: https://epub.ub.uni-muenchen.de/273/1/FB_161.pdf (Accessed November 2, 2022).

[ref54] Kozma R. B. Russell J. (1997). Multimedia and understanding: expert and novice responses to different representations of chemical phenomena. J. Res. Sci. Teach. 34, 949–968. doi: 10.1002/(SICI)1098-2736(199711)34:9<949::AID-TEA7>3.0.CO;2-U

[ref55] Kozma R. B. Russell J. (2005). “Students becoming chemists: developing representational competence” in Visualization in science education. ed. Gilbert J. K. (Berlin, Germany: Springer-Verlag), 121–146.

[ref56] Kroß A. Lind G. (2001). The impact of prior knowledge on the intensity and quality of self-explanations during studying worked-out examples from the domain of biology. Unterrichtswissenschaft 29, 5–25. doi: 10.1080/10888691.2016.1266092

[ref57] Kuhn J. Rophol M. Groß J. (2017). “Fachdidatischer Mehrwert durch Einführung digitaler Werkzeuge” in Lernprozesse mit digitalen Werkzeugen unterstützen: Perspektiven aus der Didaktik naturwissenschaft licher Fächer. eds. Meßinger-Koppelt J. Schanze S. Groß J. (Hamburg, Germany: Joachim Herz Stiftung), 11–12.

[ref58] Kultusministerkonferenz (2005). Bildungsstandards im Fach Chemie für den Mittleren Schulabschluss. Available at: http://www.kmk.org/fileadmin/Dateien/veroeffentlichungen_beschluesse/2004/2004_12_16-Bildungsstandards-Chemie.pdf

[ref59] Lienert G. A. Raatz U. (1998). Testaufbau und Testanalyse (6. Auflage). Weinheim, Germany: Beltz Psychologie Verlags Union.

[ref60] Lind G. Friege G. Kleinschmidt L. Sandmann A. (2004). Beispiellernen und Problemlösen. Zeitschrift für Didaktik der Naturwissenschaften 10, 29–49. doi: 10.1007/s40573-020-00104-w

[ref61] Lind G. Friege G. Sandmann A. (2005). Selbsterklären und Vorwissen. Zeitschrift zur Theorie und Praxis erziehungswissenschaftlicher. Forschung 19, 1–29. doi: 10.1026/1617-6391/a000421

[ref62] Mayer R. E. (2002). Multimedia learning. Ann. Rep. Educ. Psychol. Jpn. 41, 27–29. doi: 10.5926/arepj1962.41.0_27

[ref63] Mayer R. E. (2014). “Cognitive theory of multimedia learning” in The Cambridge handbook of multimedia learning. ed. Mayer R. E.. 2nd ed (Cambridge, United Kingdom: Cambridge University Press), 43–71.

[ref64] Mayer R. E. Moreno R. (1998). A split-attention effect in multimedia learning: evidence for dual processing systems in working memory. J. Educ. Psychol. 90, 312–320. doi: 10.1037/0022-0663.90.2.312

[ref65] Mayer R. E. Moreno R. (2002a). Aids to computer-based multimedia learning. Learn. Instr. 12, 107–119. doi: 10.1016/S0959-4752(01)00018-4

[ref66] Mayer R. E. Moreno R. (2002b). Animation as an aid to multimedia learning. Educ. Psychol. Rev. 14, 87–99. doi: 10.1023/A:1013184611077

[ref67] Mayring P. (2010). Qualitative Inhaltsanalyse. Grundlagen und Techniken. 2nd Edn. Weinheim: Beltz-Verlag.

[ref68] Milgram P. Kishino F. (1994). A taxonomy of mixed reality visual displays. IEICE transactions on. Inf. Syst. E77-D(12), 1321–1329.

[ref69] Nerdel C. (2017). Grundlagen der Naturwissenschaftsdidaktik-Kompetenzorientiert und aufgabenbasiert für Schule und Hochschule. Heidelberg: Springer-Spektrum.

[ref70] Osterlind S. J. (1998). Constructing test items: Multiple-choice, constructed-response, performance, and other formats (2. Aufl.). Evaluation in education and human services: Boston, MA, USA: Kluwer Academic Publishers.

[ref71] Peeters H. Habig S. Fechner S. (2023). Does augmented reality help to understand chemical phenomena during hands-on experiments?–implications for cognitive load and learning. Mult. Technol. Interact. 7, 1–17. doi: 10.3390/mti7020009

[ref9001] Prümper J. (2008). ISONORM 92441/110-S. Beurteilung von Software auf Grundlage der Internationalen Energie-Norm DIN EN ISO. 9241–110.

[ref72] Radu I. Schneider B. (2019). “What can we learn from augmented reality (AR)?” in Proceedings of the 2019 CHI conference on human factors in computing systems (S. 1–12). eds. Brewster S. Fitzpatrick G. Cox A. Kostakos V. (New York, NY, USA: ACM).

[ref73] Reid N. (2021). The Johnstone triangle. Cambridge, United Kingdom: Royal Society of Chemistry.

[ref74] Reinfried S. Mathis C. Ulrich N. (2009). Das Modell der Didaktischen Rekonstruktion. Eine innovative Methode zur fachdidaktischen Erforschung und Entwicklung von Unterricht. Beiträge zur Lehrerbildung 27, 404–414.

[ref75] Reinhold F. (2019). Wirksamkeit von Tablet-PCs bei der Entwicklung des Bruchzahlbegriffs aus mathematikdidaktischer und psychologischer Perspektive: Wiesbaden, Germany: Springer Fachmedien Wiesbaden.

[ref76] Rodić D. D. Rončevi T. N. Segedinac M. N. (2018). The accuracy of macro–submicro–symbolic language of future chemistry teachers. Acta Chim. Slov. 65, 394–400. doi: 10.17344/acsi.2017.4139, PMID: 29993106

[ref9002] Rost J. (1998). Drei Thesen zum Konzept qualitativer Forschungsmethoden. In: Zeitschrift für die Didaktik der Chemiewissenschaften.

[ref77] Rost J. (2004). Lehrbuch Testtheorie-Testkonstruktion. 2nd Edn. Psychologie Lehrbuch: Huber.

[ref78] Sailer M. Murböck J. Fischer F. (2017). Digitale Bildung an bayerischen Schulen–Infrastruktur, Konzepte, Lehrerbildung und Unterricht. München: vbw.

[ref79] Sandmann A. (2014). “Lautes Denken – die Analyse von Denk-, Lern-und Problemlöseprozessen” in Methoden in der naturwissenschaftsdidaktischen Forschung. eds. Krüger D. Parchmann I. Schecker H. (Berlin Heidelberg: Springer), 179–188.

[ref80] Sandmann A. Hosenfeld M. Mackensen I. Lind G. (2002). “Paraphrasieren, Schlussfolgern, Bewerten-Strategien des Lernens mit Beispielaufgaben bei Experten und Novizen in Biologie” in Lehr-und Lernforschung in der Biologiedidaktik. eds. Klee R. Bayrhuber H. (Innsbruck, Austria: StudienVerlag), 131–144.

[ref81] Santos V. C. Arroio A. (2016). The representational levels: influences and contributions to research in chemical education. J. Turkish Sci. Educ. 13, 3–18. doi: 10.12973/tused.10153a

[ref82] Scheerer F. (2021). Interaktion mit der MBP IoT-Plattform mittles der Microsoft Holo Lens [Masterarbeit]. Stuttgart: Universität Stuttgart.

[ref83] Schmalhofer V. R. (1996). The effects of biotic and abiotic factors on predator-prey interactions in old-field flower-head communities: School of Graduate Studies, Rutgers The State University of New Jersey.

[ref84] Schmalstieg D. Höllerer T. (2016). Augmented reality–principles and practice. Boston: Addison-Wesley.

[ref85] Schnitker J. (2016). Das Unsichtbare sichtbar machen – Chemie lehren mit Simulationen auf der Teilchenebene. 6. Ausg Edn. Neuss: Klaus Seeberger Verlag.

[ref86] Schnotz W. (2001a). Lernen aus Beispielen: Ein handlungstheoretischer Rahmen (Kommentar). Unterrichtswissenschaft 29, 88–95. doi: 10.1080/10691898.2018.1534895

[ref87] Schnotz W. (2001b). Wissenserwerb mit Multimedia. Unterrichtswissenschaft 29, 292–318. doi: 10.1016/j.learninstruc.2014.04.003

[ref88] Schnotz W. (2014). “An integrated model of text and picture comprehension” in The Cambridge handbook of multimedia learning. ed. Mayer R. E.. 2. Aufl., S. (Cambridge, United Kingdom: Cambridge University Press), 72–103.

[ref9003] Schnotz W. (2005). An Integrated Model of text and Picture Comprehension. In: R.E. Mayer (Hg.): The Cambridge Handbook of Multimedia Learning. 2. Aufl. New York: Cambridge University Press. 2, 49–70.

[ref9004] Schnotz W. (2011). Pädagogische Psychologie. Weinheim: Beltz-Verlag.

[ref89] Schnotz W. Bannert M. (2003). Construction and interference in learning from multiple representation. Learn. Instr. 13, 141–156. doi: 10.1016/S0959-4752(02)00017-8

[ref90] Schwanke H. Trefzger T. (2020). “Augmented Reality in Schulversuchen der E-Lehre in der Sekundarstufe I. In DPG (Vorsitz)” in Didaktik Der Physik-Beiträge Zur DPG-Frühjahrstagung (Bonn, Germany: Deutsche Physikalische Gesellschaft (DPG)).

[ref91] Simon B. (2001). E-learning an Hochschulen. Gestaltungsräume und Erfolgsfaktoren von Wissensmedien: Josef Eul Verlag.

[ref92] Slater M. Wilbur S. (1997). A framework for immersive virtual environments (FIVE): speculations on the role of presence in virtual environments. Presence Teleoperat. Virtual Environ. 6, 603–616. doi: 10.1162/pres.1997.6.6.603

[ref93] Staatsinstitut für Schulqualität und Bildungsforschung. (2023a). Ergänzende Informationen zum Lehrplan PLUS: Gymnasium, Chemie, Jahrgangsstufen 8 (NTG), 9 (SG, MuG, WSG) - Häufig beobachtbare Fehlvorstellungen zum Teilchenkonzept. Munich, Germany: Staatsinstitut für Schulqualität und Bildungsforschung.

[ref9008] Staatsinstitut für Schulqualität und Bildungsforschung. (2023b). Ergänzende Informationen zum LehrplanPLU. Grundschule, Heimat- und Sachunterricht, Jahrgangsstufen 1/2 - Das Stoff-Teilchen-Konzept.

[ref94] Sweller J. (2011). “Cognitive load theory” in Psychology of learning and motivation. The psychology of learning and motivation: Cognition in education. eds. Mestre J. P. Ross B. H., vol. 55 (Amsterdam, Netherlands: Elsevier), 37–76.

[ref95] Sweller J. Chandler P. Tierney P. Cooper M. (1990). Cognitive load and selective attention as factors in the structuring of technical Cooper113material. J. Exp. Psychol. Gen. 119, 176–192. doi: 10.1037/0096-3445.119.2.176

[ref96] Sweller J. van Merrienboer J. J. G. Paas F. G. W. C. (1998). Cognitive architecture and instructional design. Educ. Psychol. Rev. 10, 251–296. doi: 10.1023/A:1022193728205

[ref97] Taber K. S. (2013). Revisiting the chemistry triplet: drawing upon the nature of chemical knowledge and the psychology of learning to inform chemistry education. Chem. Educ. Res. Pract. 14, 156–168. doi: 10.1039/C3RP00012E

[ref98] Talanquer V. (2011). Macro, submicro, and symbolic: the many faces of the chemistry “triplet”. Int. J. Sci. Educ. 33, 179–195. doi: 10.1080/09500690903386435

[ref99] Tepner O. Dollny S. (2014). “Entwicklung eines Testverfahrens zur Analyse fachdidaktischen Wissens” in Methoden in der naturwissenschaftsdidaktischen Forschung. eds. Krüger D. Parchmann I. Schecker H. (Berlin Heidelberg: Springer), 311–323.

[ref100] Terzer E. Hartig J. Upmeier zu Belzen A. (2013). Systematische Konstruktion eines Tests zu Modellkompetenz im Biologieunterricht unter Berücksichtigung von Gütekriterien. Zeitschrift für Didaktik der Naturwissenschaften 19, 51–76.

[ref101] Thees M. Kapp S. Strzys M. P. Bell F. Lukowicz P. Kuhn J. (2020). Using Smartglasses as an efficient augmented reality tool for enhancing university STEM laboratory courses—theoretical derivations and empirical findings from a multimedia learning perspective. Comput. Hum. Behav. 102, 104130. doi: 10.1016/j.chb.2019.06.020

[ref102] Thyssen C. Hoffmann C. Probst C. Huwer J. (2020). Augmented Reality - unterrichten mit erweiterter Realität. NiU Biol. 455, 41–44. doi: 10.1007/s11576-019-00956-9

[ref103] Treagust D. F. Chittleborough G. Mamiala T. (2003). The role of submicroscopic and symbolic representations in chemical explanations. Int. J. Sci. Educ. 25, 1353–1368. doi: 10.1080/0950069032000070306

[ref104] Tricot A. Sweller J. (2014). Domain-specific knowledge and why teaching generic skills does not work. Educ. Psychol. Rev. 26, 265–283. doi: 10.1007/s10648-013-9243-1

[ref105] Tschiersch A. Krug M. Huwer J. Banerji A. (2021). ARbeiten mit erweiterter Realität im Chemieunterricht – ein Überblick über Augmented Reality in naturwissenschaftlichen Lehr-Lernszenarien. CHEMKON 28, 241–244. doi: 10.1002/ckon.202100009

[ref106] Van Dijk T. A. Kintsch W. (1983). Strategies of discourse comprehension. New York, NY, USA: Academic Press.

[ref107] Van Driel J. H. Verloop N. (2002). Experienced teachers’ knowledge of teaching and learning of models and modelling in science education. Int. J. Sci. Educ. 24, 1255–1272. doi: 10.1080/09500690210126711

[ref108] Venkatesh V. Davis F. D. (2000). A theoretical extension of the technology acceptance model: four longitudinal field studies. Manag. Sci. 46, 186–204. doi: 10.1287/mnsc.46.2.186.11926

[ref109] Vosniadou S. (1994). Capturing and modeling the process of conceptual change. Learn. Instr. 4, 45–69. doi: 10.1016/0959-4752(94)90018-3

[ref110] Walpuski M. Ropohl M. (2014). “Statistische Verfahren für die Analyse des Einflusses von Aufgabenmerkmalen auf die Schwierigkeit” in Methoden in der naturwissenschaftsdidaktischen Forschung. eds. Krüger D. Parchmann I. Schecker H. (Berlin Heidelberg: Springer), 385–398.

[ref111] Wirtz M. A. (2013). Dorsch–Lexikon der Psychologie. 6th Edn. Bern: Hans Huber.

[ref112] Wolf M. Söbke H. (2020). Augmented Reality in der Hochschullehre für Bauingenieure: Ein leichtgewichtiger Ansatz. Beitrag im Hochschulforum Digitalisierung. Available at: https://hochschulforumdigitalisierung.de/de/blog/augmented-reality-bauingenieurwesen (Accessed November 2, 2022).

[ref113] Wyss C. Bührer W. Furrer F. Degonda A. Hiss J. A. (2021). Innovative teacher education with the augmented reality device Microsoft Holo Lens—results of an exploratory study and pedagogical considerations. Mult. Technol. Interact. 5:45. doi: 10.3390/mti5080045

[ref114] Wyss C. Furrer F. Degonda A. Bührer W. (2022). Augmented Reality in der Hochschullehre. MedienPädagogik 47, 118–137. doi: 10.21240/mpaed/47/2022.04.06.X

[ref115] Zhao F. Schnotz W. Wagner I. Gaschler R. (2020). Texts and pictures serve different functions in conjoint mental model construction and adaption. Mem. Cogn. 48, 69–82. doi: 10.3758/s13421-019-00962-0, PMID: 31372846

